# Sex-dependent alterations in the physiology of entorhinal cortex neurons in old heterozygous 3xTg-AD mice

**DOI:** 10.1186/s13293-020-00337-0

**Published:** 2020-11-16

**Authors:** Dany Arsenault, Cyntia Tremblay, Vincent Emond, Frédéric Calon

**Affiliations:** 1 Faculty of Pharmacy, Université Laval, Quebec City, QC Canada; 2 Neuroscience, Centre de Recherche du CHU de Québec (CHUQ), Quebec City, QC Canada; 3 Physiotek, Quebec City, QC Canada

**Keywords:** Alzheimer, Aging, Entorhinal cortex, 3xTg-AD mice, Electrophysiology

## Abstract

While the higher prevalence of Alzheimer’s disease (AD) in women is clear, studies suggest that biological sex may also influence AD pathogenesis. However, mechanisms behind these differences are not clear. To investigate physiological differences between sexes at the cellular level in the brain, we investigated the intrinsic and synaptic properties of entorhinal cortex neurons in heterozygous 3xTg-AD mice of both sexes at the age of 20 months. This brain region was selected because of its early association with AD symptoms. First, we found physiological differences between male and female non-transgenic mice, providing indirect evidence of axonal alterations in old females. Second, we observed a transgene-dependent elevation of the firing activity, post-burst afterhyperpolarization (AHP), and spontaneous excitatory postsynaptic current (EPSC) activity, without any effect of sex. Third, the passive properties and the hyperpolarization-activated current (Ih) were altered by transgene expression only in female mice, whereas the paired-pulse ratio (PPR) of evoked EPSC was changed only in males. Fourth, both sex and transgene expression were associated with changes in action potential properties. Consistent with previous work, higher levels of Aβ neuropathology were detected in 3xTg-AD females, whereas tau deposition was similar. In summary, our results support the idea that aging and AD neuropathology differentially alter the physiology of entorhinal cortex neurons in males and females.

## Background

The prevalence of Alzheimer’s disease (AD) is higher for women than men. While this difference is mainly explained by the gap in longevity, there is also evidence of disparity in pathological processes between sexes. For example, AD pathology is more strongly associated with clinical dementia in women than in men [1–5]. The importance of risk factors is also dependent on sex, with midlife diabetes and APOE ε4 allele even more strongly associated with AD in women [6–8]. Moreover, a postmortem study revealed that women exhibit greater senile plaque deposition at early stages of neurofibrillary tangle development [9]. Accumulated data thus suggest that women may display a higher vulnerability to the disease. On the other hand, it is not clear whether these differences involve functional changes at the cellular level during neural development or a loss of neuroprotection by female hormones after menopause [10–12].

The triple-transgenic model of AD (3xTg-AD) displays Aβ plaques, tau-laden neurofibrillary tangles, and age-dependent alterations in memory function and was developed to investigate both canonical markers of AD neuropathology in the same animal [13–16]. Studies performed in 3xTg-AD mice consistently report higher Aß burden in females [17–19] and sex-dependent disturbance of social behaviors [19], a less characterized behavioral symptom of dementia [20, 21]. Interestingly, Bories et al. reported biphasic alterations (social disinhibition followed by social apathy) in 3xTg-AD mice occurring 6 months earlier in females [19], which is in agreement with a higher susceptibility to AD/dementia in women [19]. Moreover, this study noted no direct relationship between social dysfunctions and Aß/tau pathologies. In counterpart, the authors found that the sex- and age-dependent behavioral alterations observed in 3xTg-AD mice coincided with changes in basal synaptic activity of the medial prefrontal cortex, a brain region known to be critical for mediating social behavior [22–25]. Another study reported that female 3xTg-AD mice displayed a significant deterioration in glucose tolerance compared to their male counterparts [18]. Energy failure is also known to play a key role in AD-related brain network hyperactivity in the APP/PS1 mouse model of AD [26]. The impact of metabolic dysfunction on brain functions may thus be more important in females, adding another explanation behind the physiological alterations that could influence differently AD progression, brain function, and/or pathological behavior in both sexes. Another study showed that the earlier performance decline of 3xTg-AD females observed in cognitive tasks is associated with an enhanced corticosterone response [14]. Finally, difference in sexual hormones between males and females is a factor known to modulate neuronal function [27] and AD neuropathology [10], suggesting a possible link between both factors. Thus, these results suggest that physiological changes at the cellular and molecular level are key factors to explain the sex differences in the development of clinical symptoms.

Entorhinal cortex (EC) is a region known to play a key role in cognitive processes [28, 29] that also suffers a significant loss of neurons during the first stages of AD [30]. Neurofibrillary tangles, a pathological hallmark of AD, are observed primarily in the EC in mild AD and then apparently spread to the hippocampus and other cortical areas as the disease progresses [31, 32]. It has been hypothesized that AD originates in the EC because APP expression was found to be higher in EC compared to other cortical areas in cognitively intact people [33]. In mice, a study showed that a limited transgenic expression of APP/Aß to EC and subiculum induces learning and memory deficits [34], supporting the idea that this brain region is a key structure in AD-related cognitive decline. Our laboratory has previously shown a decline of cognitive functions in homozygous 12-month-old 3xTg-AD mice and intracellular recordings revealed that this behavioral dysfunction was associated with some abnormalities in the physiology of layer 5 EC neurons. For example, we identified an increase in spontaneous excitatory postsynaptic events (sEPSC), an elevation of the firing activity (output), and some changes in action potential (AP) properties [35]. No significant sex differences were observed at the time, despite the large number of recorded cells. However, we could not conclude to an absence of sex difference for two main reasons. First, these data compared neuronal physiology at only one age period. The development of AD neuropathologies in 3xTg-AD mice is much more pronounced in the EC than the frontal cortex [16, 36]. Consequently, it is possible that compensatory mechanisms in the EC in response to genetically programmed development of Aß and tau pathologies have already been exceeded at 12 months of age, hiding a potential gender difference. Supporting this hypothesis, a study performed in 3xTg-AD mice showed earlier cognitive impairment in females (before 12 months of age) [14]. Second, physiological/natural development of late-onset AD also includes aging processes and it is possible that the greater susceptibility of women to this neurodegenerative disorder involves a synergy between pathological factors and senescence mechanisms in neurons. The alterations of intrinsic properties as compensatory mechanisms during aging have been previously documented in a review by Rizzo [37], supporting the idea that gender differences could involve a synergy of both factors.

The goal of this study was therefore to investigate sex, age, and transgene expression as three independent variables affecting physiological properties of EC neurons from 3xTg-AD mice. Heterozygous (rather than homozygous) mice were used to lessen the impact of the genetic component of the model in order to not “overflow” the effects of sex difference and aging. Moreover, experiments were performed at 20 months of age, i.e., in animals 8 months older than in a previous study using homozygotes [35], to ensure sufficient pathology development. Our hypothesis was that the use of a less aggressive model of Aß/tau pathologies, while giving more weight to mechanisms of cellular aging, could unmask sex differences at the functional level. These conditions should include/amplify mechanisms of neuronal senescence while maintaining a slower genetically programmed development of AD, expectedly reducing the risk of saturating mechanisms of cellular compensation.

## Methods

### Ethics approval

All experiments were approved by the Laval University Animal Care and Use Committee in accordance with the standards of the Canadian Council on Animal Care.

### Transgenic model

Animals were produced and maintained in the animal facilities of the Research Center of *Institut Universitaire en Santé Mentale de Québec* at 22 ± 1 °C under a 12-h light/dark cycle regime. Water and food were available ad libitum. The 3xTg-AD mouse model has been described previously [16, 35, 36, 38, 39]. These transgenic mice develop an age-related progressive neuropathological phenotype that includes both plaques and tangles distributed along a regional pattern similar to AD [36, 38, 40, 41]. Finally, this AD mouse model presents behavioral and cognitive changes that are correlated with the development of Aβ and tau pathologies [42]. Nontransgenic (NonTg) mice were derived from the original mouse line and were of the same genetic background. Experiments were performed only in heterozygous mice and both females and males were used in this study.

### Preparation of tissue samples

All experiments were performed with the same animals. The right hemisphere was devoted for electrophysiology studies. The left hemisphere was quickly dissected and the parietotemporal cortex was assigned for Western immunoblots. Molecular analyses included the parietal cortex, in addition to the temporal cortex in order to obtain a sufficient quantity of tissue for all biochemical experiments. Tissue extracts (50 mg of mouse tissue) were homogenized in 8 volumes of Tris-buffered saline (TBS) containing phosphatase inhibitors (1 mM each of sodium vanadate and sodium pyrophosphate, 50 mM sodium fluoride), protease inhibitors (Complete), 10 μg/ml pepstatin A, and 0.1 mM EDTA (Sigma-Aldrich). Samples were sonicated briefly (3 × 10 s) and centrifuged at 100,000*g* for 20 min at 4 °C, and supernatants were collected to generate TBS-soluble intracellular/extracellular fractions (soluble fractions). The TBS-insoluble pellets were sonicated in 8 volumes of lysis buffer (150 mM NaCl, 10 mM NaH2PO4, 1% Triton X-100, 0.5% SDS, and 0.5% deoxycholate) containing the same cocktail of protease and phosphatase inhibitors. The resulting homogenates were centrifuged at 100,000*g* for 20 min at 4 °C and supernatants were collected to produce lysis-buffer soluble fractions (detergent-soluble or membrane fractions). Final pellets were homogenized in 175 μl of 90% formic acid followed by sonication (3 × 10 s) to generate detergent-insoluble fractions and were divided in two aliquots that were dried out with a SpeedVac (Thermo Savant). One was solubilized in guanidine-HCl (5 M guanidine in Tris-HCl 0.05 M) and then sonicated shortly for solubilization to be used for ELISA; the other was solubilized in Laemmli’s buffer for Western immunoblotting.

### ELISA

Human amyloid 40/42 ELISA kits (Covance for soluble Aß40, Wako for insoluble Aß40 and for Aß42) were used to analyze mouse cortical tissue. Experiments were performed in soluble and insoluble protein fractions according to the manufacturers’ recommendations and the plates were read at 450 nm using a Synergy HT multidetection microplate reader (Biotek).

### Western immunoblotting

Protein concentration was determined using bicinchoninic acid assays (Pierce). For Western immunoblotting, equal amounts of protein per sample (15 μg) were added to Laemmli’s loading buffer, heated to 95 °C for 5 min before loading, and subjected to SDS-PAGE (8%). Proteins were electroblotted onto PVDF membranes (Millipore) before blocking in 5% nonfat dry milk and 1% BSA in PBS-Tween 20 for 1 h. Membranes were immunoblotted with appropriate primary and secondary antibodies followed by chemiluminescence reagents (Lumiglo Reserve; KPL). Band intensities were quantified using a Kodak Image Station 4000MM Digital Imaging System (Molecular Imaging Software version 4.0.5f7; Carestream Health). The following antibodies were used in this study: mouse anti-tau (Covance, clone tau-13, #MMS-520R-500), mouse anti-phospho-tau (Bio-Rad, clone AD2, phosphorylated at serines 396 and 404, #56484) and rabbit-glyceraldehyde-3-phosphate dehydrogenase (GAPDH; Abm, #Y413969), mouse anti-actin (ABM, #Y061021), mouse anti-drebrin (Progen Biotechnik GmbH, #GP254), rabbit anti-gephyrin (Abcam, #ab25784), rabbit anti-glutamic acid decarboxylase 65 (GAD65, Millipore, #ABN101), mouse anti GABAa receptor subunit 1 (GABAaR, Neuromab, 1:250, #75-136), mouse anti-NMDA receptor GluN2B subunit (Covance, clone n59/36, #MMS-5148-100), rabbit anti-vesicular GABA transporter (VGAT; Novus Biologicals, #NB110-55238), mouse anti-PSD-95 (NeuroMab, #75-028), mouse anti-synaptophysin (Millipore, #MAB332), mouse anti-tubulin (ABM, #G094), mouse anti-NMDA receptor subunit NR1 (NR1, advance immuno chemical, # GNR1), mouse anti-AMPA receptor GLUR2 subunit (GluR2, Neuromab, #75-002), and mouse anti-synapse-associated protein 102 (SAP102) (cloneN19/2, Neuromab, #75-058).

### Slice preparation for electrophysiology recordings

Brain slices were prepared as described previously [35, 43]. Briefly, mice were deeply anesthetized with ketamine (100 mg/kg, i.p.) and xylazine (10 mg/kg, i.p.) and decapitated. The brain was removed quickly (< 60 s) and placed in an ice-cold solution containing the following (in mM): 210 sucrose, 3.0 KCl, 0.75 CaCl_2_, 3.0 MgSO_4_, 1.0 NaH_2_PO_4_, 26 NaHCO_3_, and 10 glucose saturated with 95% O_2_/5% CO_2_. Horizontal slices of 250 μm were cut from inferior to superior brain with a vibrating tissue slicer (VT 1000s; Leica) and kept at room temperature in ACSF containing the following (in mM): 124 NaCl, 3.0 KCl, 1.5 CaCl_2_, 1.3 MgCl_2_, 1.0 NaH_2_PO_4_, 26 NaHCO_3_, and 20 glucose saturated with 95% O_2_/5% CO_2_. Slices were allowed to recover for at least 1 h before recording. A slice was then transferred to a chamber exposed to ACSF flowing at a rate of 2–3 ml/min. Recordings were performed between 30 and 32 °C.

### Whole-cell patch clamp recording

The slices were viewed first with a 4× objective and the deep layer of the EC was located beside the hippocampus. For most animals, two to three slices were recorded per hemisphere. Large deep layer neurons in the EC were then viewed under near-infrared illumination with a 40× water-immersion objective (Fluor, 40×, 0.80 W; Nikon) and a charge-coupled device camera (IR-1000; Dage MTI). Patch pipettes were pulled from thick-walled borosilicate glass (1.5/0.84 mm; WPI) on a horizontal puller (P-97; Sutter Instruments). The pipette solution contained the following (in mM): 100 KMeSO_4_, 15 KCl, 4 ATP-Mg, 10 creatine phosphate, 10 HEPES, 0.1 EGTA, pH 7.2, adjusted with KOH, and 275–280 mOsm. Electrodes had resistances between 5 and 7 MΩ. The seal resistance was > 2 GΩ. Whole-cell recordings were made at the soma with a Multiclamp 700A amplifier (Molecular Devices). The access resistance, usually between 20 and 50 MΩ, was monitored throughout each experiment and only recordings with stable access were used. Experiments were conducted using pClamp 9.2 (Molecular Devices). Data were digitized at 8 kHz and were not filtered, except for the sEPSC protocol where the recordings were filtered at 1 kHz and digitized at 16 kHz.

### Data analysis for electrophysiology experiments

Passive and active properties (firing rate and afterhyperpolarization (AHP) potential) were tested in I-clamp, whereas hyperpolarization-activated cation (Ih) current, sEPSC, and evoked excitatory postsynaptic currents (eEPSC) were quantified in V-clamp. The electrophysiological analyses were performed using Clampfit 9.2 (Molecular Devices). Cell conductance (Gc) was estimated from the slope of the graph of hyperpolarizing current injection (*I*) versus voltage variation (*V*). The calculation was derived from the equation *I* = Gc*V. The injected current duration was 400 ms and hyperpolarized current amplitudes were 50, 100, 150, and 200 pA. Cell capacitance (CC) (i.e., for a first-order resistance-capacitance circuit) was estimated from the linear slope of the plot of I*T = CC*V, where *T* is the time constant of voltage variation (as measured by fitting a single-exponential function for a voltage decay over time, *V* = *V*α (1 − *e*^−*T*/(R*CC)^), where *R* is the input resistance (i.e., Gc^−1^) and *V*α is the asymptote, so that *t* = RC (i.e., *V* = 0.632 *V*α), using a graphical method) [35, 44]. We measured CC in the *I*-clamp configuration because this recording mode generated more accurate values than *V*-clamp [45]. However, the methodologies used in this study were not isopotential and they did not exclude contaminations by dendritic processes or by the presence of slow voltage-dependent phenomena [46]. Although the presence of these two phenomena is minimal in the voltage range used to evaluate our parameters, the estimations of Gc/resistance/CC made in this study should be interpreted as relative rather than absolute values. The firing rate was estimated by counting the number of spikes during the 3-s current step and the result was plotted versus the amplitude of the injected current (F-I graph). The slope of the F-I plot was calculated for the firing frequencies included between 0 and 15 Hz by linear regression. Firing adaptivity was estimated by comparing intervals between APs at the beginning and the end of the train of APs generated by the injection of a current. The minimum current necessary to elicit an action potential (so-called rheobase [47]) was estimated from the F-I plot using graphical methods. APs were detected using a threshold of voltage. The AP amplitude and the undershoot were calculated relative to the threshold [48]. The post-spike hyperpolarization was calculated from the stationary period of the undershoot to the peak of the transient hyperpolarization, following an AP (Fig. 7b) [35, 49]. Post-burst AHP potential was characterized following a burst of AP generated by 50-ms current step. The peak amplitude was compared to the resting potential whereas decay time was estimated by fitting a single-exponential function. Ih-channel activity was measured in voltage-clamp mode as the amplitude of the slowly activating inward current component elicited by 1-s voltage steps from − 60 to − 100 mV in 10-mV increments. Excitatory and inhibitory inputs were discriminated by generating postsynaptic currents at different imposed voltages (Fig. 11a, b). The intensity of electrical stimulation was between 10 and 400 μA (during 50 μs) and was applied at 0.1 Hz between each episode. The interval between electrical stimulations in paired protocol stimulation was 100 ms (i.e., 10 Hz). Excitatory inputs produced a depolarizing current that increases with the hyperpolarization of resting potential while inhibitory inputs generated both depolarizing and hyperpolarizing currents, depending on the imposed voltage. We observed a hyperpolarizing current for potentials greater than the reversal potential of Cl^−^ ions, estimated at − 63 mV using the Nernst equation, whereas an inhibitory input produced a depolarizing current under this potential. Short-term plasticity was evaluated by calculating the paired-pulse ratio (second peak amplitude divided by first peak amplitude, PPR) for a paired electrical stimulation of 100-ms intervals. The sEPSCs were automatically detected using the event detection package of Clampfit 9.2 (Molecular Devices). This package uses multiple pre-established templates to optimize the detection of synaptic events.

### Statistical analysis

Values are expressed as mean +/SEM. Normality of distribution was assumed for each group. Statistical comparisons were performed using a two-way ANOVA for the study of two variables simultaneously. When variable interaction was detected, statistical comparisons between groups were performed depending on the variance equivalence between groups. Groups of data that failed tests for equal variance were analyzed by Welch’s *t* test between animals of the same sex but different genotype (sex-dependent effect) or between males and females of the same genotype (transgene-dependent effect). An unpaired Student’s *t* test was performed to compare groups of equal variance. When only two groups were compared, unpaired Student’s *t* tests were carried out, except for unequal variance, for which a Welch’s *t* test was used. Finally, the coefficient of determination (*r*^2^) and the significance of the degree of linear relationship between various parameters were determined with a simple regression model. Statistical analyses were performed using JMP statistical analysis software (version 8.0.2).

## Results

### Female 3xTg-AD mice showed more pronounced Aß pathology without significant change in insoluble tau deposits

**Fig. 1:**
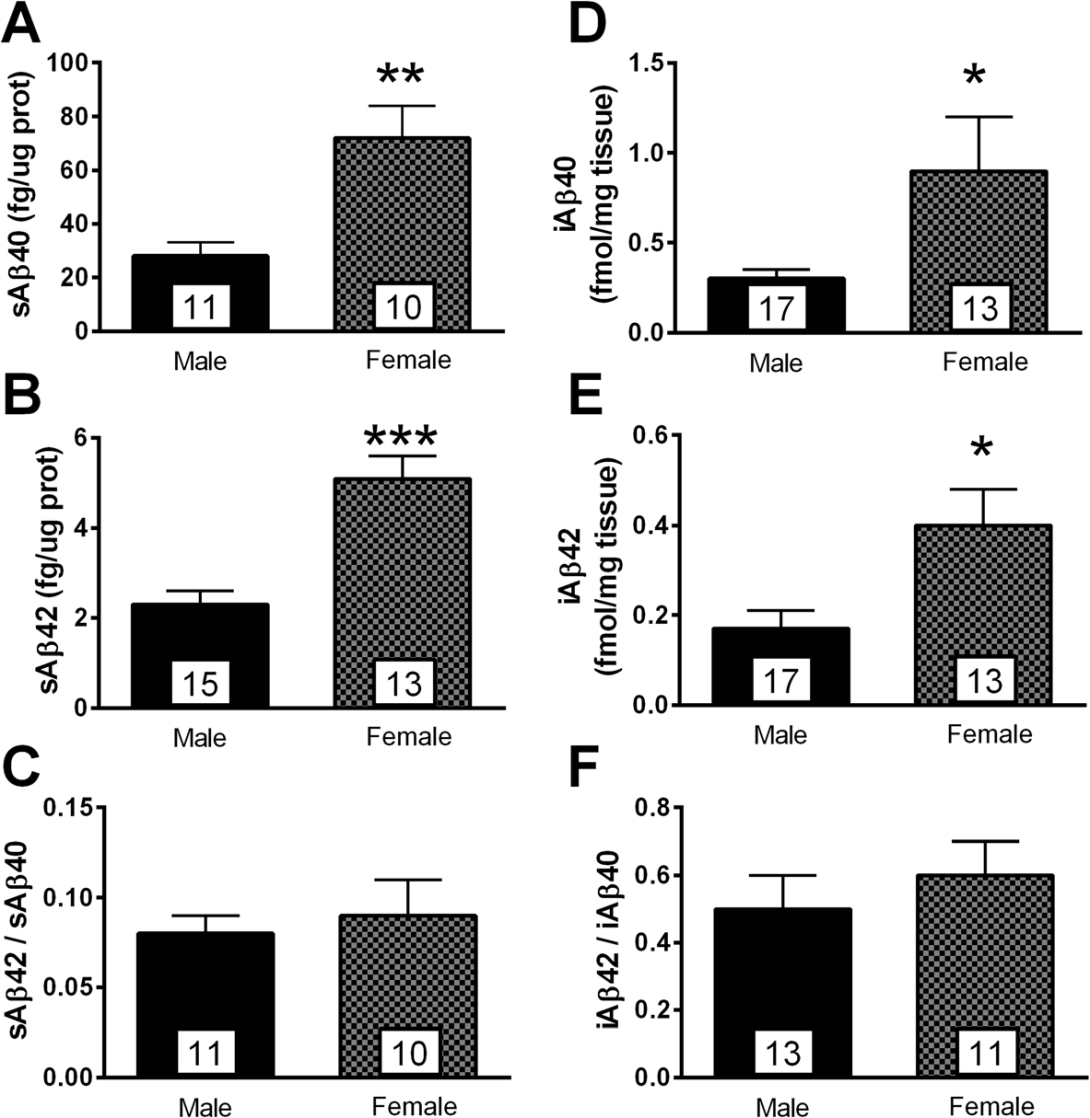
Aβ pathology was more pronounced in 20-month-old heterozygous 3xTg-AD females. **a**, **b** Levels of both sAβ40 and sAβ42 were higher in transgenic females compared to males. **c** The sAβ42/sAβ40 ratio was not influenced by sex. **d**, **e** In insoluble fractions, amounts of iAβ40 and iAβ42 were more elevated in transgenic females. **f** The sex of animals did not modulate the iAβ42/iAβ40 ratio in insoluble fractions. Statistical comparisons were performed using Welch’s *t* test (**a**, **d**, and **e**) or unpaired Student’s *t* test (**b**, **c**, and **f**). *p < 0.05, **p < 0.01, ***p < 0.001

**Fig. 2:**
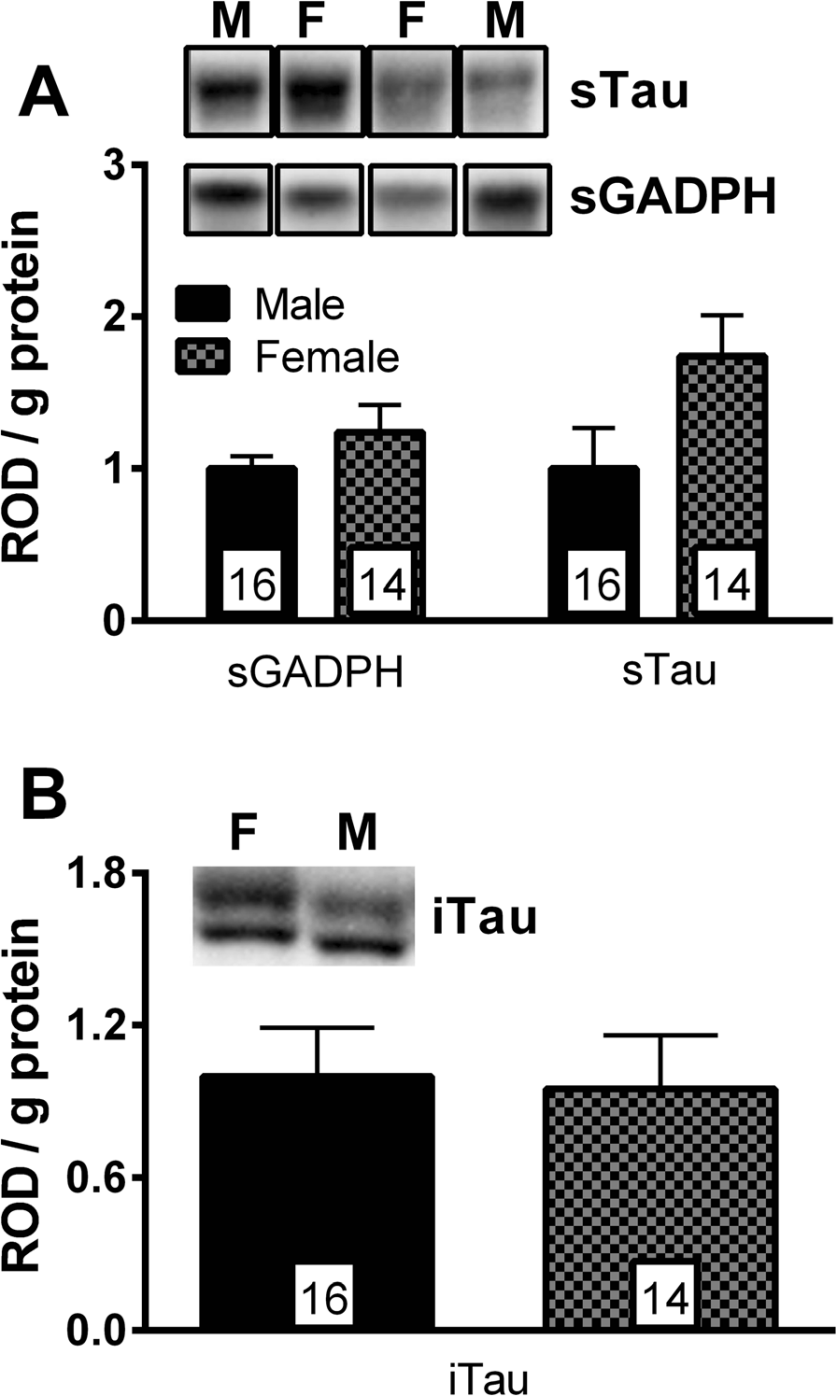
Sex did not influence tau pathology in 20-month-old 3xTg-AD mice. **a** Amounts of GADPH and tau in soluble fractions were similar between males and females. **b** No difference between the sexes was found in the tau levels and the proportion of phosphorylated tau at serine 396/404 in insoluble fractions. Statistical comparisons were performed using unpaired Student’s *t* test. Abbreviations: ROD, relative optical value; GADPH, glyceraldehyde-3-phosphate dehydrogenase (used as control)

Studies performed in 3xTg-AD mice have reported differences according to biological sex, particularly for behavior and accumulation of Aß [17–19]. To investigate the basis of this sexual dimorphism, we first quantified molecular factors associated with Aß and tau pathologies in the parietotemporal cortex of 20-month-old heterozygous 3xTg-AD mice. As expected, we observed a more pronounced Aß pathology in both soluble (Aß40: *t* = − 3.51, *p* = 0.0024; Aß42: *t* = − 4.91, *p* < 0.001) and insoluble fractions (Aß40: *t* = − 2.22, *p* = 0.035; Aß42: *F* = 6.23, *p* < 0.023) from female 3xTg-AD mice (Fig. 1). On the other hand, sex difference in the amount of tau in soluble (*t* = 1.90, *p* = 0.074) and insoluble (*t* = 0.178, *p* = 0.430) fractions did not reach statistical significance (Fig. 2).

### Sex-dependent alterations of passive properties by 3xTg-AD expression

**Fig. 3:**
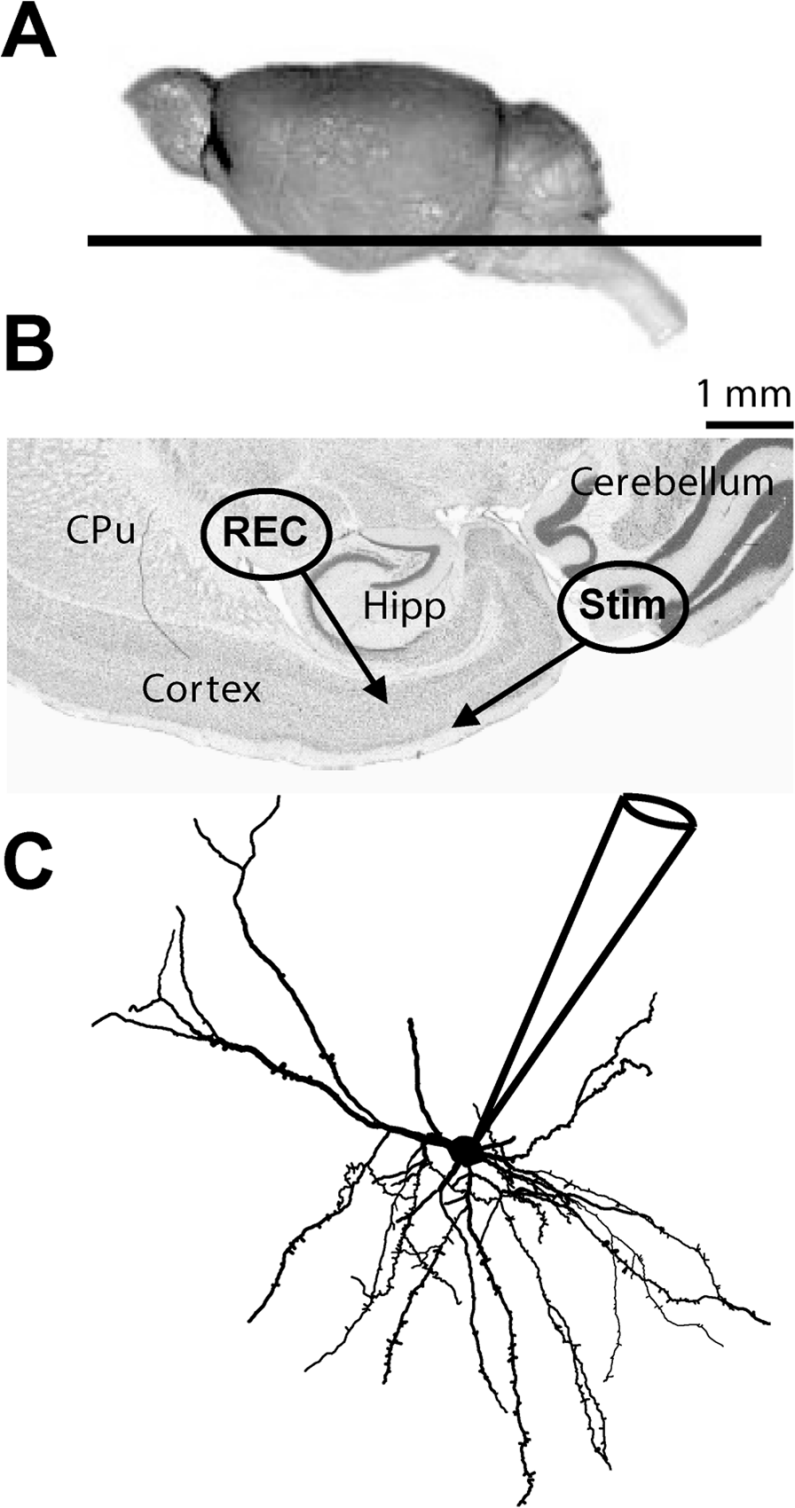
Tissue preparation for electrophysiological recordings and dietary treatment. **a** Side view of the mouse brain. The black line represents the 250 μm horizontal section used in this study. **b** Horizontal mouse brain section stained with hematoxylin nuclear counterstain. Whole-cell recordings (REC) were made in the deep layer of EC. **c** Whole-cell patch-clamp recordings of deep-layer EC neurons. Abbreviations: CPu, caudate putamen (striatum); EC, Entorhinal cortex; Hipp, hippocampus; Rec, patch clamp cell recording region; Stim, electrically stimulated region

**Fig. 4:**
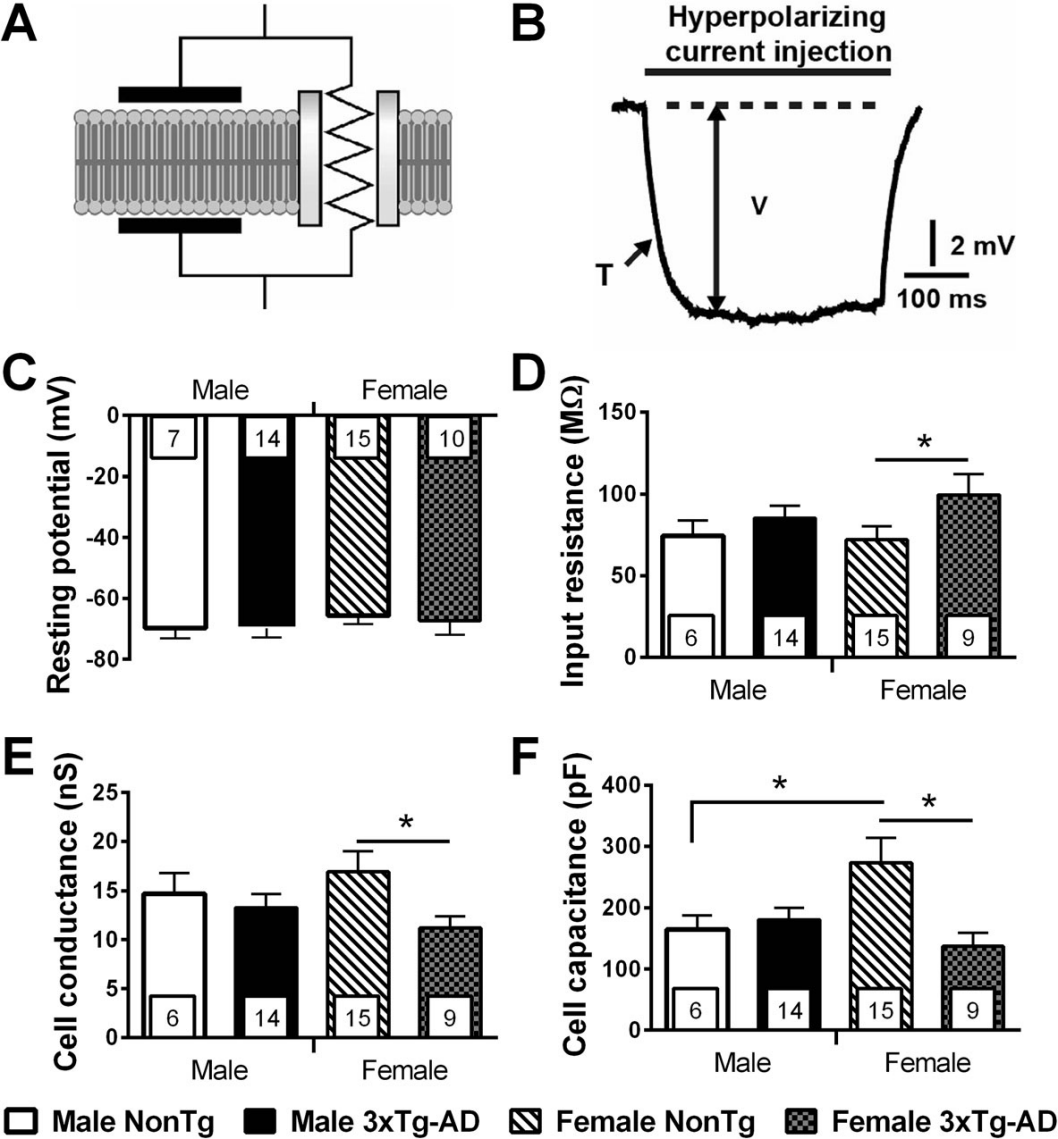
Transgene expression changed the passive properties of EC deep-layer neurons only in 20-month-old females. **a** Electrical representation of a cell membrane. **b** To quantify passive properties, different intensities of hyperpolarizing current were injected into a neuron in current clamp: voltage variation (V) and time constant (T) were measured after each injection. The panel illustrated on one trace the electrical properties measured for the calculation of passive properties. **c** The resting potential was not influenced by transgenes or sex. The expression of AD-related transgenes increased input resistance (**d**) and reduced Gc (**e**) in neurons of female animals, but not in males. (**f**) CC of neurons in NonTg females was higher than these of 3xTg-AD females and NonTg males. The number of mice included in each group was 4 for NonTg males, 5 for 3xTg-AD males, 8 for NonTg females, and 4 for 3xTg-AD females. Statistical comparisons were performed using unpaired Student’s *t* tests (**c**, **d**, and **e**) or Welch’s *t* tests (**f**). Abbreviations: CC, cell capacitance; Gc, cell conductance. *p < 0.05

Using a patch-clamp approach (Fig. 3), we investigated the passive properties (Fig. 4a) of layer 5 EC neurons by injecting hyperpolarizing current (Fig. 4b). First, the resting potential was not affected by the sex of animals (Student’s *t* test, NonTg: *t* = 0.778/*p* = 0.441, 3xTg-AD: *t* = − 0.492/*p* = 0.625) or by transgene expression (Student’s *t* test, males: *t* = − 0.009/*p* = 0.943; females: *t* = 0.363/*p* = 0.718) (Fig. 4c). Second, transgene expression increased the input resistance (Fig. 4d) and decreased the Gc (Fig. 4e) only in females (Student’s *t* test, *t* = − 2.09, *p* = 0.043 for the input resistance; *t* = 2.24, *p* = 0.030 for Gc). Third, we observed a higher CC in NonTg females, compared to female 3xTg-AD (Welch’s *t* test, *F* = 8.81, *p* = 0.007) or to NonTg males (Welch’s *t* test, *F* = 5.55, *p* = 0.029). In addition, the CC correlated with levels of insoluble tau (*r*^2^ = 0.5327, *p* = 0.009, *N* = 23). In accordance with this latter observation, our previous work showed an inverse relationship between the CC and the phosphorylation of tau in 12-month-old homozygous 3xTg-AD mice [35], supporting a link between this electrophysiological property and tau pathology.

### Transgene expression was associated with increased firing activity of EC in both sexes

**Fig. 5:**
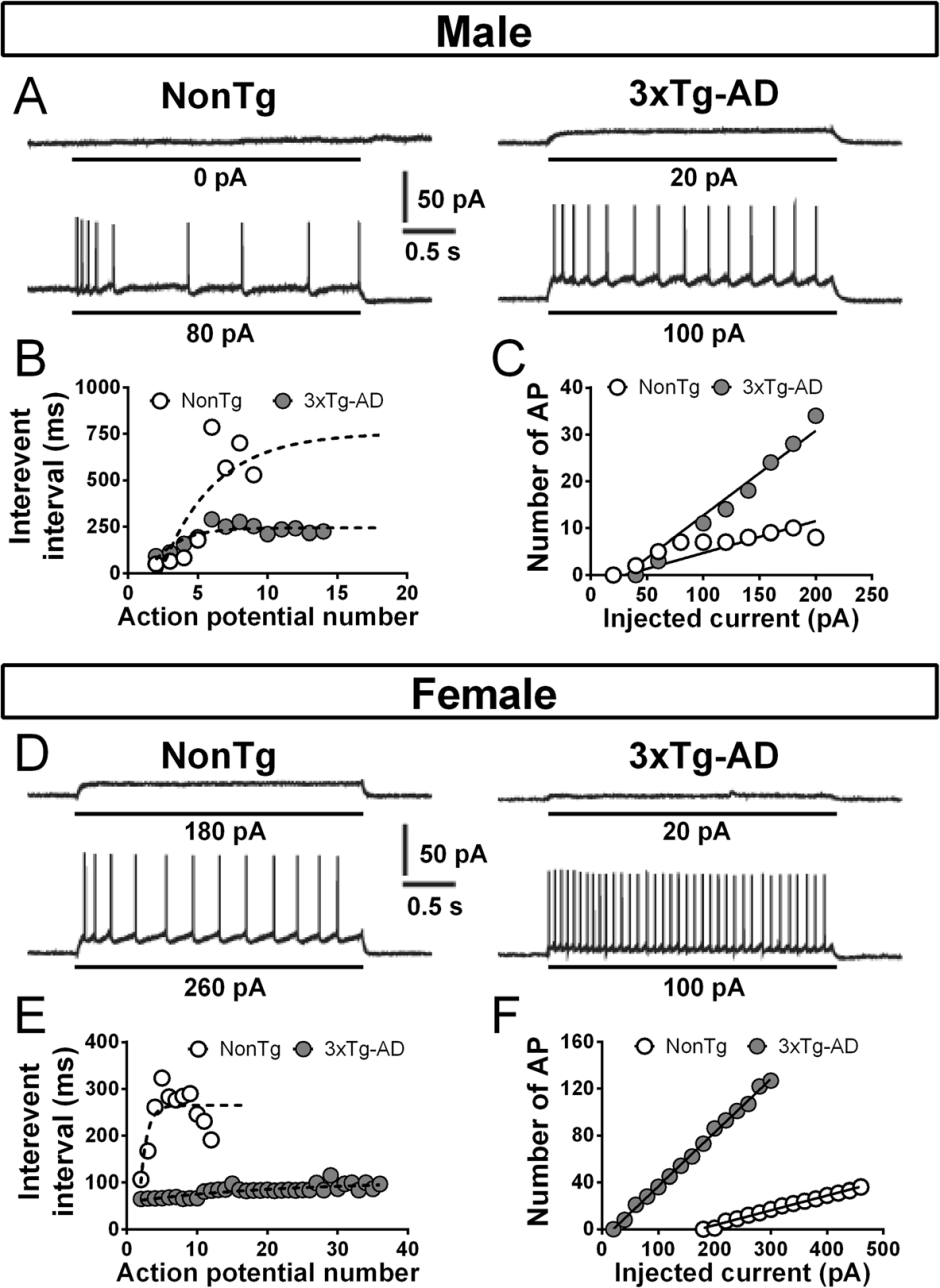
Examples of electrophysiological recordings showing the firing properties of EC neurons, accordingly to the sex and the genotype. **a** The upper trace illustrates the maximum current injected in a neuron without reaching its excitation threshold. The lower trace represents the firing pattern obtained for a current injection of 80 pA greater than that of the upper trace in the same neuron. The protocol used is a 3-s depolarizing current injection generating a voltage response. Left recording shows the firing of a neuron from a NonTg male while a cell from a transgenic male is illustrated in the one at right. **b** Interevent interval between action potential of the recordings presented in the panel **a**. The firing accommodation corresponds to the difference between interevent interval at the beginning and the end of the train. 3xTg-AD neurons showed a lower firing adaptivity compared to NonTg cells. **c** The relationship between firing rate and injected current (F-I curves) from NonTg or 3xTg-AD neurons of males is illustrated in the graph on the right of the panel. The steepness of F-I slopes was increased by transgene expression in males. **d**, **e**, and **f** Same as **a**, **b**, and **c**, but it is for females. Transgene expression influenced similarly the firing activity and the firing accommodation in neurons of female mice

**Fig. 6:**
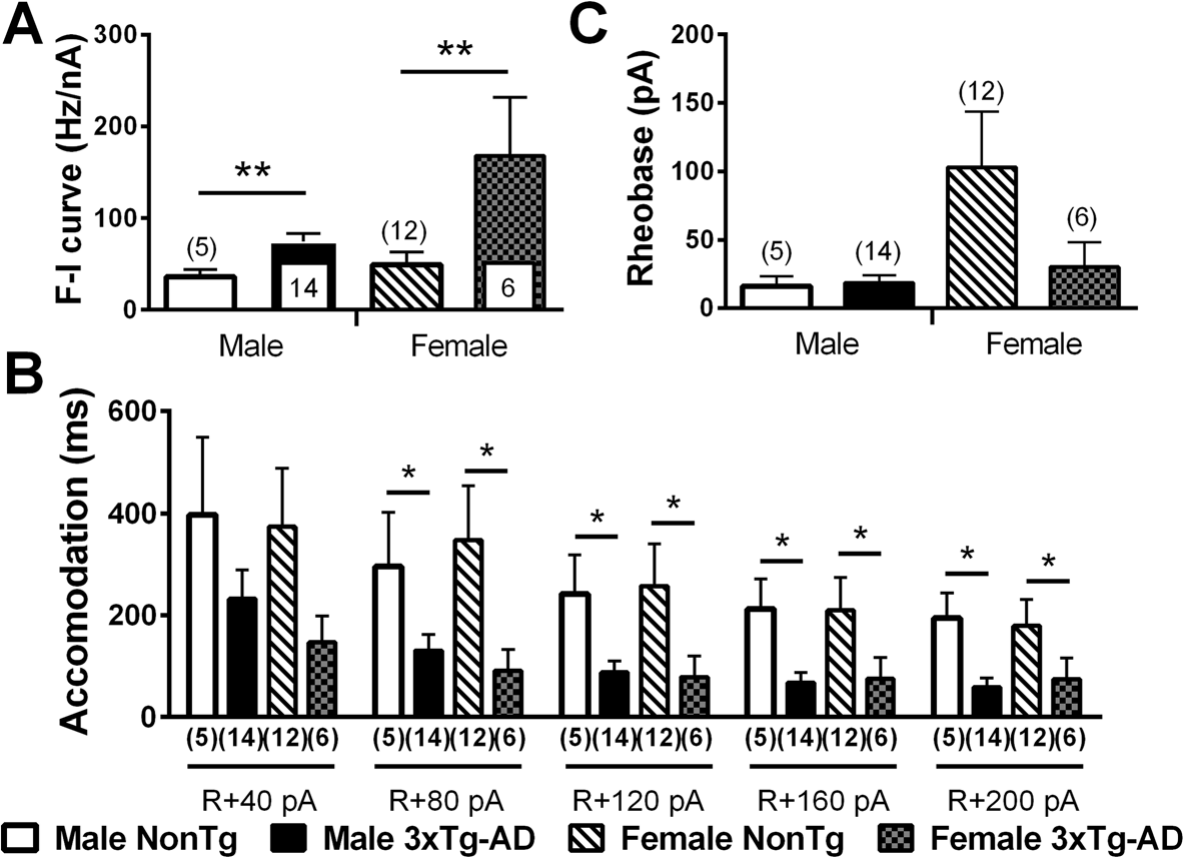
Transgene expression increased firing activity and reduced firing accommodation in both males and females aged of 20 months. Transgene expression increased F-I slopes (**a**) and reduced firing accommodation (**b**) in 3xTg-AD mice of both sexes. **c** The rheobase was not influenced by 3xTg-AD expression or sex and was estimated from the F-I plot using graphical methods. The number of mice included in each group was 5 for NonTg males, 14 for 3xTg-AD males, 12 for NonTg females, and 6 for 3xTg-AD females. Statistical comparisons were performed using two-way ANOVA (**a**, **b**) or Welch’s *t* test (**c**). Abbreviations: F-I, firing rate versus injected current. *p < 0.05, **p < 0.01

The input-output relationship notifies about how neurons code information in the brain. To investigate this electrophysiological property, we performed steps of depolarizing current (input) and quantified three fundamental features of the firing activity transmitted by neurons: the “Firing rate-Injected current (F-I)” curves, the intensity of depolarization required to deliver an AP (rheobase), and the accommodation (Fig. 5). We found higher F-I curves (two-way ANOVA, genotype: *F*(1) = 12.75/*p* = 0.001, Fig. 6a) and a lower accommodation (two-way ANOVA, genotype: *F*(1) = 5.71/*p* = 0.023 for current of + 80 pA over the maximum current injected in a neuron without reaching its excitation threshold, *F*(1) = 6.05/*p* = 0.019 for current of + 120 pA, *F*(1) = 6.53/*p* = 0.015 for current of + 160 pA, *F*(1) = 7.05/*p* = 0.012 for current of + 200 pA, Fig. 6b) in 3xTg-AD mice of both sexes. The graphically calculated rheobase was not significantly different between each group (Fig. 6c). A positive relationship was observed between F-I curves and insoluble Aß42 (*r*^2^ = 0.5674, *p* = 0.03, *N* = 19). This transgene-dependent increase of firing activity was in accordance with what we previously reported in 12-month-old 3xTg-AD mice [35].

### Sex and transgene expressions were altering AP properties differently

**Fig. 7:**
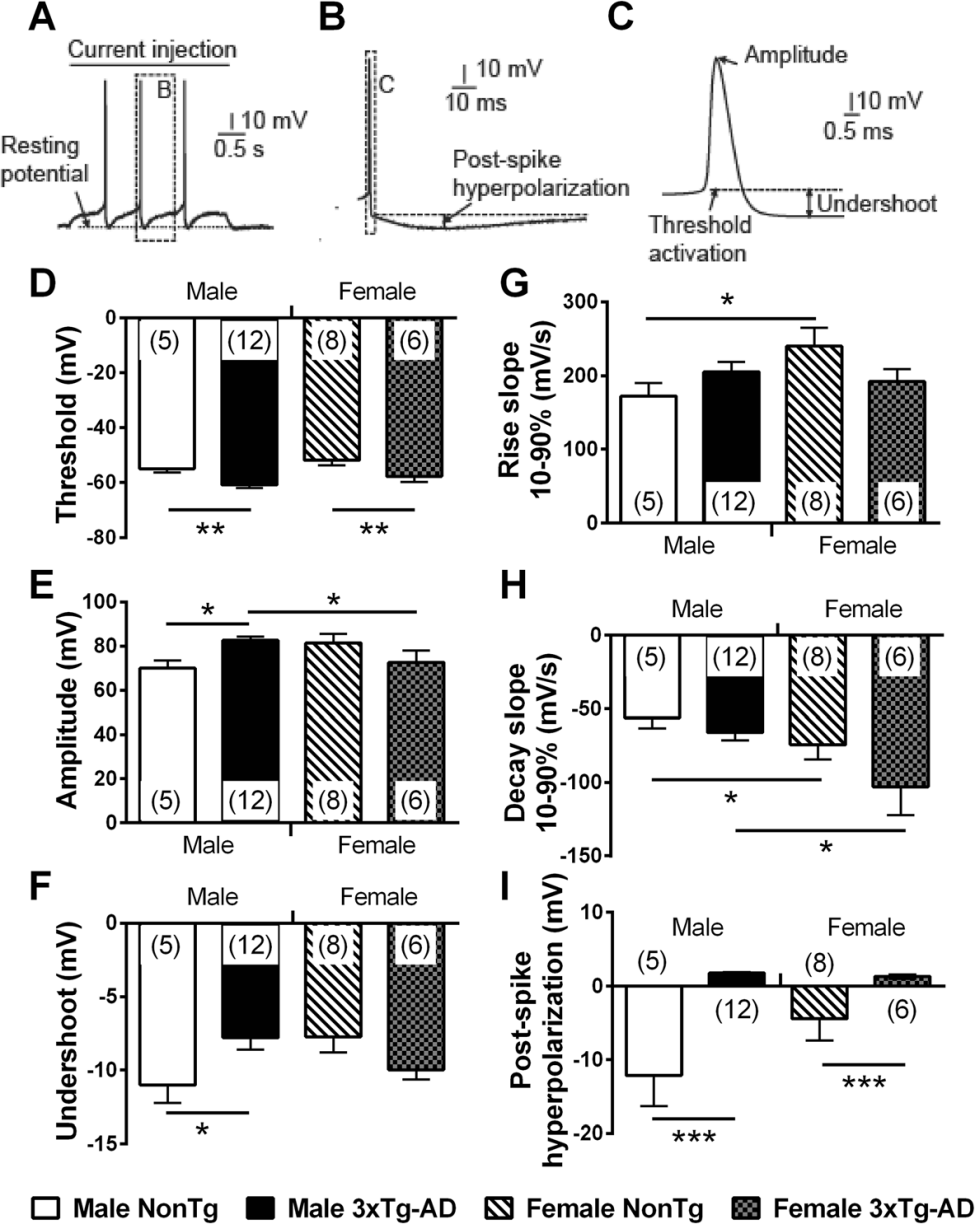
AP properties are differently influenced by sex and transgene expression in EC from 20-month-old mice. **a** An example of a recorded EC neuron following an injection of a 3-s depolarizing current. In this typical trace, the injected current triggered three APs. **b** Representation of a post-spike hyperpolarization (zoomed from the dashed square in **a**). Post-spike hyperpolarization was calculated from the difference between the voltage undershoot after the AP (the dashed line) and the voltage peak of post-spike. **c** Representation of AP characteristics quantified in this study (zoomed from the dashed square in the panel **b**). Undershoot was the difference between stabilized voltage after the AP and activation threshold. **d** AP threshold was significantly decreased with transgene expression in both sexes. **e** Amplitude of AP was higher in neurons from male 3xTg-AD, compared to those from male NonTg and female 3xTg-AD. **f** Transgene expression reduced undershoots only in the male. The rising slope was lower in NonTg males (**g**) whereas decay slope was higher in both NonTg and 3xTg-AD male mice (**h**). Transgene expression reduced post-spike hyperpolarization in both sexes. The number of mice included in each group was 5 for NonTg males, 12 for 3xTg-AD males, 8 for NonTg females, and 6 for 3xTg-AD females. Statistical comparisons were performed using two-way ANOVA (**d**, **h**, and **i**) or unpaired Student’s *t* test (**e**, **f**, and **g**). Abbreviations: AP, action potential; EC, entorhinal cortex. *p < 0.05, **p < 0.01, ***p < 0.001

AP is the electrical unit used by neurons to communicate. Our laboratory previously demonstrated that a change in this signal impacts the synaptic response detected by postsynaptic neurons [50], confirming the key role of AP in brain function. In addition, modulation of AP properties is reported during learning processes [51] and aging [37]. To investigate if the conditions used in this study modulated AP properties, we quantified key characteristics associated with a single AP (Fig. 7a–c). Firstly, we observed a decrease in the threshold of APs in 3xTg-AD neurons of both sexes (two-way ANOVA, genotype: *F*(1) = 12.75/*p* = 0.001, Fig. 7d). Similar results were reported in neocortical layer II/III pyramidal cells from APP transgenic model of AD [52]. Secondly, we found a lower amplitude (Fig. 7e) and a higher undershoot (Fig. 7f) in neurons from 3xTg-AD males compared to NonTg animals of the same sex (Student’s *t* test, *t* = − 2.48/*p* = 0.019 for amplitude and t = − 2.27/*p* = 0.031 for undershoot). These changes demonstrate a gain in hyperpolarizing currents making up the AP in comparison with depolarizing ones. Thirdly, the rising slope was faster in female NonTg neurons compared to male NonTg cells (Student’s *t* test, *t* = 2.28/*p* = 0.030, Fig. 7g) and the females of both genotypes showed a higher decay slope than males (two-way ANOVA, genotype: *F*(1) = 6.55/*p* = 0.016, Fig. 7h). Finally, the post-spike AHP was lower in NonTg neurons versus 3xTg-AD neurons in both males and females (two-way ANOVA, genotype: *F*(1) = 21.51/*p* < 0.001, Fig. 7I). We also observed a negative association between the level of insoluble Aß42 and the post-spike AHP (*r*^2^ = − 0.5174, *p* = 0.0334, *N* = 17). Interestingly, these sex-dependent changes in amplitude and undershoot of AP were not reported in homozygous 3xTg-AD mice aged of 12 months [35], showing that specific conditions are necessary to discriminate a difference of sex in these two parameters.

### Transgene expression modulated the post-burst AHP differently between males and females

**Fig. 8:**
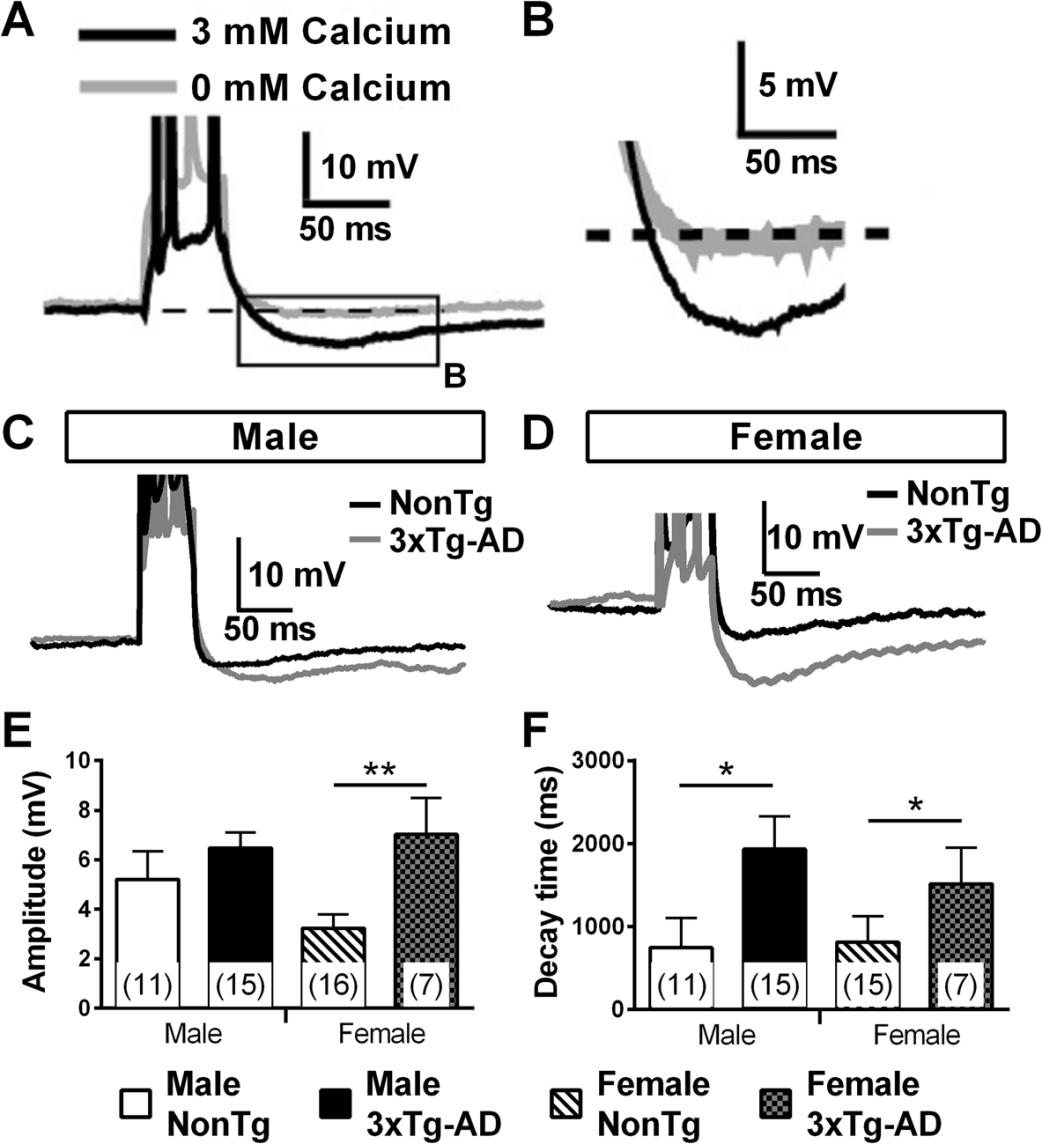
Transgene expression modulated the post-burst AHP potential in 20-month-old mice. **a** An example of a recorded EC neuron following an injection of a 50-ms depolarizing current. Post-burst AHP potential is estimated in relation with the resting potential (the dashed line) and is abolished when calcium is removed from the extracellular solution. **b** Representation of a post-burst hyperpolarization (zoomed from the square in **a**). **c** An example of recordings illustrating post-burst hyperpolarization in neurons from male (**c**) or female (**d**) expressing or not 3xTg-AD transgenes. Transgene expression increased the amplitude of post-burst AHP only in females (**e**) and elevated the decay time in both sexes (**f**). The number of mice included in each group was 5 for NonTg males, 5 for 3xTg-AD males, 9 for NonTg females, and 4 for 3xTg-AD females. Statistical comparisons were performed using two-way ANOVA (**f**) or unpaired Student’s *t* test (**e**). *p < 0.05, **p < 0.01

Post-burst AHP potential is known to play a key role in cognitive function by modulating neuronal excitability during learning processes [53–56]. In addition, many studies reported an amplification of this current during aging [54–57] and in AD [58]. To investigate this current, we triggered 2–3 APs by injecting 50 ms depolarizing current and quantified the peak and the decay time of the AHP potential. This current was abolished if calcium was removed from the extracellular solution (Fig. 8a, b). We observed an elevation of the amplitude with transgene expression only in females (Student’s *t* test, *t* = 2.83/*p* = 0.007, Fig. 8c–e), whereas the decay time was longer in 3xTg-AD neurons of both sexes (two-way ANOVA, genotype: *F*(1) = 5.70/*p* = 0.021, Fig. 8c, d, and f). This current has never been quantified in previous studies using 3xTg-AD mice.

### Sex-dependent reduction of Ih current by transgene expression

**Fig. 9:**
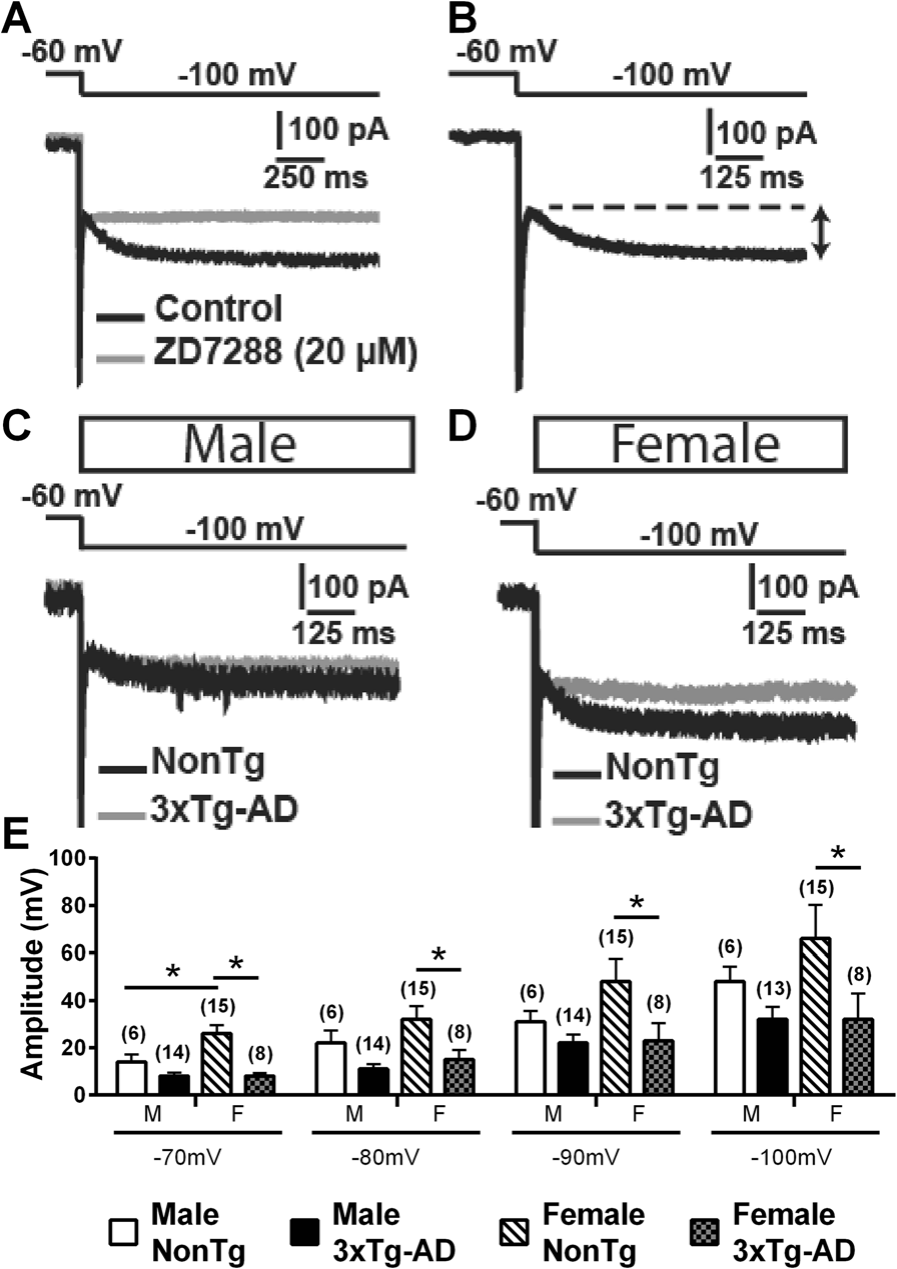
Transgene expression reduced Ih current in a sex-dependent manner in mice aged of 20 months. **a** An example of recorded neuron following a voltage step, from − 60 to − 100 mV. Application of ZD7288 (20 μM), an antagonist of the hyperpolarized-activated current Ih [57], in the same neuron showed its slow and persistent activation in EC. **b** Ih was measured by subtracting the current before and after its slow and persistent activation, as illustrated by the line with two arrows. Illustrations of Ih currents generated by a voltage step, from − 60 to − 100 mV in males (**c**) and females (**d**), both transgenic or NonTg animals. **e** Ih current generated by hyperpolarizing voltage steps was decreased by 3xTg-AD expression in females, whereas the reduction was not significant in males. The number of mice included in each group was 4 for NonTg males, 5 for 3xTg-AD males, 8 for NonTg females, and 4 for 3xTg-AD females. Statistical comparisons were performed using unpaired Student’s *t* test (− 80 to − 100 mV) or Welch’s *t* test (− 70 mV). *p < 0.05

HCN proteins are subunits known to generate the Ih channel [59]. A previous study reported that HCN1 can form a complex with APP in the murine brain and levels of this protein are significantly reduced in the brains of sporadic AD patients compared with age-matched healthy subjects [60]. In addition, overexpression of HCN1 in Neuro2a cells decreases Aβ generation, whereas blockage of Ih channel activity restores the level of Aβ production [60]. Put together, these data suggest a role of HCN in AD. In this study, we investigated the current generated by HCN channels (Ih) in deep-layer EC neurons from 3xTg-AD mice. The current generated by this channel was quantified by a protocol including steps of hyperpolarized voltage (from − 60 to − 70/− 80/− 90/− 100 mV) and an application of the antagonist ZD7288 (20 μM) [61] blocked the current (Fig. 9a, b). We observed a lower Ih current in female 3xTg-AD neurons when compared to NonTg neurons from the same sex (Welch’s *t* test for step to − 70 mV, *F* = 19.7/*p* = 0.0003, Student’s *t* test for other steps, *t* = − 2.58/*p* = 0.012 for step to − 80 mV, *t* = − 2.24/*p* = 0.031 for step to − 90 mV, *t* = − 2.03/*p* = 0.049 for step to − 100 mV, Fig. 9c–e). Moreover, males showed a lower Ih current than females for a voltage step from − 60 to − 70 mV in NonTg animals (Welch’s *t* test, *F* = 6.14/*p* = 0.024, Fig. 9e). Finally, the level of sAß42 and iAß40 negatively correlated with the Ih current generated by a step from − 60 to − 80 mV (*r*^2^ = − 0.6368, *p* = 0.014, *N* = 14 for sAß40; *r*^2^ = − 0.4557, *p* = 0.043, *N* = 20 for iAß40), supporting the link between Aβ pathology and Ih current.

### Basal excitatory synaptic activity was increased by transgene expression in both sexes

**Fig. 10:**
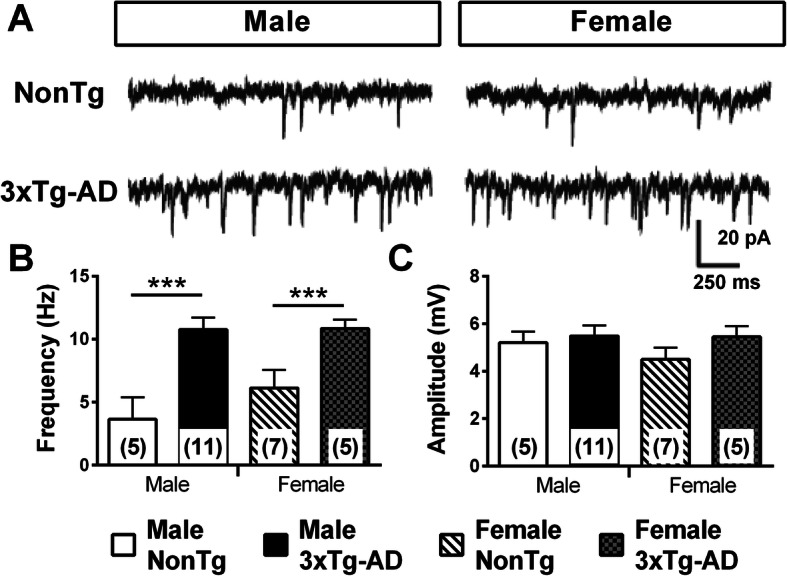
Transgene expression increased spontaneous excitatory postsynaptic current (sEPSC) in both sexes. **a** Examples of intracellular sEPSC recordings (voltage clamped at − 60 mV). **b** Frequency of sEPSC was higher in neurons from 20-month-old 3xTg-AD mice for both sexes. **c** sEPSC amplitude was not affected by sex or transgene expression. The number of mice included in each group was 3 for NonTg males, 5 for 3xTg-AD males, 5 for NonTg females, and 4 for 3xTg-AD females. Statistical comparisons were performed using two-way ANOVA. ***p = 0.001

Brain hyperactivity and defective network activity were reported in transgenic models of AD neuropathology [34, 52, 62] and in AD patients [63–65]. We previously quantified the sEPSC of EC neurons and found that these from 12-month-old homozygous 3xTg-AD mice displayed more sEPSC than NonTg without any sex effect [35], which supports the idea of a persistent hyperactivity of glutamatergic synapses in AD. In this study, we reinvestigated the sEPSC in older heterozygous 3xTg-AD mice. Our observations were similar to those earlier obtained, that is an increase in the number of excitatory postsynaptic events by transgene expression with no effect of sex (two-way ANOVA, genotype: *F*(1) = 20.95/*p* < 0.001, sex: *F*(1) = 0.99/*p* = 0.329, Fig. 10). A positive relationship was observed between the frequency of sEPSC and insoluble Tau (*r*^2^ = 0.5392, *p* = 0.031, *N* = 16).

### Short-term plasticity of cortico-cortical excitatory input was modulated in male 3xTg-AD mice, but not in females

**Fig. 11:**
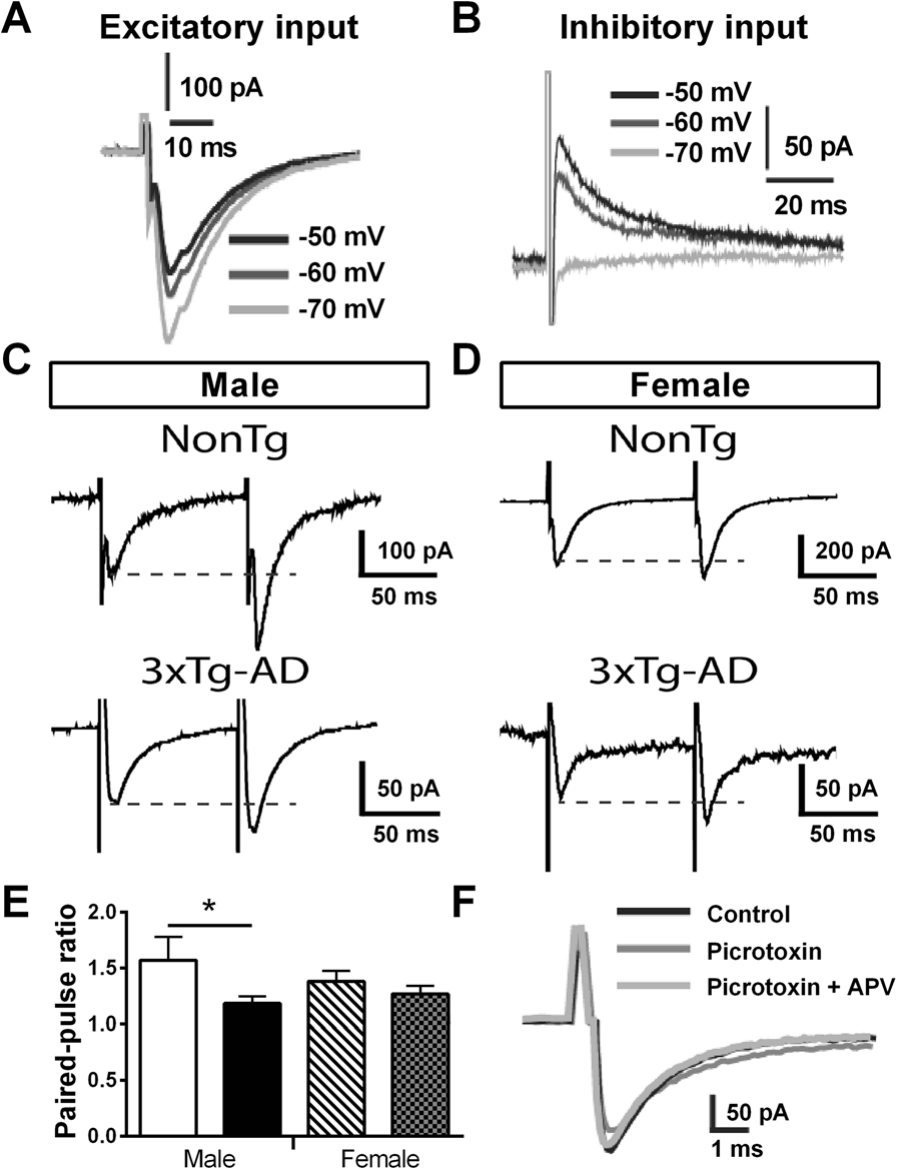
Sex-dependent alteration of paired-pulse ratio from intracortical synaptic transmission by 3xTg-AD expression in mice aged of 20 months. **a**, **b** Evoked excitatory and inhibitory input has been discriminated by generating postsynaptic currents at different imposed voltages. The excitatory inputs produced a depolarizing current that increases with hyperpolarization of resting potential, whereas inhibitory inputs generated both hyperpolarizing (− 50 mV and -60 mV) and depolarizing currents (− 70 mV), depending on whether imposed voltage was under or over the reversal potential of Cl^−^ ions, estimated at − 63 mV. There are examples of eEPSC recordings for a paired electrical stimulation (interval of 100 ms) in males (**c**) and females (**d**), both transgenic or NonTg animals. **e** The P2/P1 ratio was decreased by transgene expression in male, but not in female mice. **f** The application of the GABA_a_ receptor antagonist picrotoxin (100 μM) and the NMDA receptor antagonist D-APV (100 μM) did not affect the amplitude or the kinetic of eEPSC, showing that these receptors did not play a significant part in it. The number of mice included in each group was 3 for NonTg males, 4 for 3xTg-AD males, 4 for NonTg females, and 4 for 3xTg-AD females. Statistical comparisons were performed using unpaired Student’s *t* test. Abbreviations: eEPSC, evoked postsynaptic current. *p < 0.05

Postsynaptic responses are crucial electrophysiological properties of neurons and depend on how APs are regulated [50, 66–68]. When two bursts of AP activate synaptic transmission in a short period, the second postsynaptic response can be larger or smaller than the first. The ratio of the amplitude from the second response to that of the first is called PPR (Paired-pulse ratio) and depends on the probability of vesicular release at the synapse [69]. Then, PPR is used to measure the release probability of cortico-cortical excitatory synapses from fibers of layer 1–2 to dendrites of neurons localized in the layer 5 of EC neurons (Fig. 3c). To discriminate between excitatory and inhibitory inputs, we performed electrical stimulation at different imposed voltages (Fig. 11a, b). An excitatory input generated a depolarizing current, which increased upon the application of a hyperpolarized voltage, whereas inhibitory inputs produced a hyperpolarizing current when the imposed voltage was kept under − 63 mV (i.e., the estimated reversal potential of Cl^−^ ions) to generate a depolarizing current under that potential. Examples of PPR recorded in neurons of male and female mice expressing or not the transgenes (Fig. 11c). We found a lower PPR in male 3xTg-AD neurons, compared to NonTg cells from the same sex (Student’s *t* test, *t* = 2.70/*p* = 0.03, Fig. 11e). No difference of genotype was observed in females. Applications of GABA_a_ receptor antagonist picrotoxin (100 μM) with or without NMDA receptor antagonist D-APV (100 μM) in extracellular solution demonstrated that these two receptors were not involved in the postsynaptic current generated in this protocol (Fig. 11f). PPR negatively correlated with sEPSC (*r*^2^ = − 0.735, *p* = 0.0005, *N* = 18) and with F-I curves (*r*^2^ = − 0.5649, *p* = 0.022, *N* = 16), two factors reflecting the neuronal hyperactivity.

### Transgenic expression induced synaptic protein impairments

AD is associated with changes in synaptic proteins [35, 43, 70–75], which could have a significant impact at the cellular level. To find if sexes influenced synaptic function at the molecular level in AD, we quantified several proteins in the parietotemporal cortex by Western blot. We found an increase of SAP102 in transgenic mice of both sexes (two-way ANOVA, genotype: *F*(1) = 5.43/*p* = 0.026, Table S3). In addition, the cytosol/membrane ratio of GAD65 is increased only in transgenic females (Student’s *t* test, effect of transgenes in females: *t* = 2.79/*p* = 0.009, effect of sex in 3xTg-AD: *t* = − 2.96 /*p* = 0.006, Table S3). A positive relationship was observed between SAP102 and the cytosol/membrane ratio of GAD65 (*r*^2^ = 0.259, *p* = 0.002, *N* = 35). There was no difference for drebrin, gephyrin, VGAT, PSD-95, synaptophysin, Nr2B, NR1, NeuN, GluR2, and GABAaR (Table S3).

## Discussion

A better understanding of the biology underlying sexual divergence in AD not only could uncover clues on its pathophysiology, but also help develop more effective and personalized therapies [5, 6]. The “effects of sex differences in brain development on sex differences in brain aging, AD pathology and dementia” is among the clinical research priorities of the Society for Women’s Health Research Interdisciplinary Network on AD [6]. In agreement with this priority, the present study showed major distinctions at the cellular level, between males and females in a mouse model of AD. More specifically, we investigated the intrinsic and synaptic properties of deep-layer EC neurons in 3xTg-AD and demonstrated that aging and transgene expression affected differently their physiology, which can have an impact on the evolution of disease or its clinical expression.

### Female 3xTg-AD mice accumulate more Aß than do males

We observed higher levels of soluble and insoluble Aß40 and Aß42 in cortical tissues from 20-month-old heterozygous female 3xTg-AD mice compared to their male counterparts. This is in accordance with previous analyses of Aß pathology in the parietotemporal or frontal cortex of homozygous 3xTg-AD mice aged of 12 or 20 months [18, 19]. Another study evaluated Aß pathology in 3xTg-AD mice by a histological approach and reported a higher Aß immunoreactivity load in the frontal cortex (> 6–8 months), subiculum (> 12–14 months), and hippocampus (> 12–14 months) of females [10]. In the same study, authors showed as well that hormonal status played a key role in the difference between males and females to develop amyloid pathology [10]. In regard to tau pathology, we did not observe a statistically significant sexual divergence in the amount of soluble and insoluble tau. These results are also in agreement with previous studies performed in different brain regions of 3xTg-AD [17–19, 35], suggesting that sex has less influence on the expression of tau and its transition to an insoluble form. In sum, Aß pathology seems to be the neuropathological factor most influenced by sex.

### Physiological changes induced by transgene expression in both sexes support the hypothesis of brain network hyperactivity

Quantification of the electrical activity of a neuron is an indication of the intensity of communication between two cells. In the Tg2576 animal model of AD, the cognitive ameliorations following activation of the peroxisome proliferator-activated receptor-gamma (PPARgamma) by rosiglitazone were associated with a restoration of firing frequency in dentate gyrus cells [76], suggesting that the firing activity could be a cellular marker of AD progression. In this study, we found a higher firing activity in neurons from old heterozygous 3xTg-AD of both sexes, which was similar to what we previously observed in homozygous 3xTg-AD mice [35]. In agreement with the higher firing activity found in 3xTg-AD neurons, our results also revealed an elevation of the frequency of sEPSC in the same neurons, confirming the brain hyperactivity at the synaptic level. The higher sEPSC in old heterozygous 3xTg-AD mice was previously observed in 12-month-old homozygous mice [35] and the main suspecting mechanisms were an increased firing activity [35] and an Aβ-dependent increase of the neurotransmitter release probability [77].

Excitatory synaptic activity is known to induce rapidly the mobility of SAP102 in dendritic spines [78]. This protein plays a key role in the synaptic clearance of NMDAR [79] and is one of those involved in the regulation of inhibitory synapse formation by excitatory synaptic activity [80]. Interestingly, we observed a higher level of soluble SAP102 in transgenic animals of both sexes, confirming that brain hyperactivity found in 3xTg-AD mice impacted SAP102. We found no difference in brain levels of NMDA, suggesting that this function of SAP102 was unaltered in 3xTg-AD mice. However, our results showed a higher translocation from the cytosol to membrane of GAD65, a GABAergic presynaptic marker, only in transgenic females, suggesting that the dysregulation of SAP102 by transgenes altered inhibitory formation in a sex-dependent manner. In sum, our results support the idea of a network hyperactivity in EC of 3xTg-AD mice. At the molecular level, our results showed transgenic alteration of SAP102 in both sexes, but a dysregulation of GAD65 only in transgenic females, suggesting that females could be more susceptible to an AD-related inhibitory synaptic dysregulation.

### Potassium and sodium channels are involved in the transgenic dysregulation of neuronal excitability

To find the mechanisms behind the elevation in the firing activity of 3xTg-AD neurons, we performed additional analysis. First, we quantified AP properties and found a lower AP threshold in 3xTg-AD mice of both sexes. This property is associated with sodium channels [81] and indicated that 3xTg-AD neurons trigger more easily. In other words, it was easier for a transgenic neuron to produce brain activity than it was for NonTg neurons. A similar trend was observed previously in 12-month-old homozygous 3xTg-AD mice [35]. The amyloid cascade and the inflammatory processes induced by the development of AD pathology are two mechanisms known to modulate the activity of sodium channels and to reduce the AP threshold [82, 83]. Second, we analyzed three calcium-dependent potassium current associated with AP (or train of APs). The potassium currents are known to regulate the firing rate of neurons [51, 84–88] and could be involved in the higher firing rate found in transgenic neurons. The calcium-dependent potassium channels are divided into three main subfamilies, accordingly to their single-channel conductance [89]: SK (small conductance), IK (intermediate conductance), and BK (big conductance) channels. The BK channel is calcium- and voltage-gated potassium channels that drive AP repolarization (undershoot), and KCa1.1 is the main channel of this current in CA1 pyramidal cells [51, 90]. This current allows high-frequency firing [87, 91]. The SK and IK currents occur in response to an AP [89]. KCa2.x are the main channels of SK currents and is the main component of the post-spike hyperpolarization whereas IK currents are mainly composed of KCa3.x and represent the major component of post-train hyperpolarization [89]. The pharmacological profile differs between each of these 3 subfamilies [89, 92–95], suggesting distinct mechanisms of regulation and, possibly, different sensitivities for phenomena related to age, sex, or AD. Our results showed an abolition of the post-spike current in 3xTg-AD neurons, resulting in a strong reduction of the firing accommodation and an increase of the F-I curves. Such a reduction was previously reported in 12-month-old homozygous 3xTg-AD neurons [35]. The pathological mechanisms behind this abolition are not known. We also observed that undershoot was reduced in 3xTg-AD males, but not in females. This change is frequently associated with increased firing activity [51, 90], suggesting that males are more susceptible to neuron hyperactivity than females. On the other hand, the longer decay time (males and females) and the broader amplitude (females only) of the post-burst AHP in 3xTg-AD mice suggest that this current did not participate in the higher neuronal activity in 3xTg-AD neurons. The longer duration of this current suggested a compensatory role of the latter to the excessive firing activity observed in transgenic neurons. Post-burst AHP was also increased in hippocampal neurons of old animals presenting abnormal cognitive decline compared to control animals of the same age [57], suggesting that this physiological change could be a common cellular marker of cognitive decline.

### The physiological changes induced by transgene expression and occurring specifically in females may be an evidence of dendrite degeneration

Transgene expression induced a reduction of the Ih current in females only. Interestingly, HCN1 is a subunit of this current and a downregulation was observed in the temporal cortex of monkey during aging and in brains of sporadic AD patients compared with the brains of age-matched healthy subjects [60]. Our results revealed that old heterozygous 3xTg-AD females were more susceptible to AD-related downregulation of Ih current than their male counterparts. Neuronal activity is one of the main negative regulators of HCN1 channels and Ih current [96], suggesting that the higher firing and synaptic activities found in transgenic animals of both sexes induced a higher reduction of Ih current in females compared to males. In addition, Saito et al. demonstrated that Ih current reduced the metabolism of APP and Aß production [60]. Consequently, the lower Ih current found in transgenic females could be a factor that amplifies the production of Aß peptides in females. HCN channels in pyramidal neurons are arrayed in a gradient density pattern along the somatodendritic axis, reaching a density in the distal dendrites that is seven- to 10-fold that of the soma [97, 98]. The pharmacological inhibition of this current is known to reduce spine density and CC [99]. The membrane surface lost during the degeneration of postsynaptic spines could explain the decrease in CC. Interestingly, our results showed a similar reduction of CC in 3xTg-AD female mice, but not in 3xTg-AD male mice. In summary, our results support the idea that the lower Ih current and CC found in neurons of 3xTg-AD females reflect deregulation and deterioration of dendrites and postsynaptic spines.

## Conclusion

This study investigated the effects of biological sex in the neuronal dysfunction induced by the development of AD-like pathologies in 20-month-old heterozygous 3xTg-AD mice. It was the continuity of two previous studies performed in our laboratory. The first reported alterations of intrinsic and synaptic properties in deep layer EC neurons of 12-month-old homozygous 3xTg-AD mice, without sex difference [35]. The second described sex- and age-dependent dysfunctions of synaptic activities in frontal cortex neurons of homozygous 3xTg-AD mice [19]. The hypothesis of the present work was that different experimental conditions (accentuating aging processes) could unmask sex differences in the alterations of EC neurons driven by transgene expression, as was observed in neurons of the frontal cortex. Here, we report sex-dependent alterations of intrinsic and synaptic properties (passive, AP, Ih, post-burst AHP, PPR) in older animals with less aggressive AD neuropathologies (heterozygous rather than homozygous mice). However, sex did not modify the effect of transgene expression on firing activities and sEPSC frequency, indicating that these transgenic alterations are independent of the sex. The present research (1) confirms sex differences in neuronal changes induced by Aβ/tau-producing 3xTg-AD transgenes expression in the EC, (2) supports the idea of a higher vulnerability of EC neurons to AD in females, and (3) provides evidence that age-related factors differently affect the physiology of neurons between males and females. This work adds to the bulk of data showing studies using transgenic models of AD should monitor for sex differences when possible. In sum, the confirmation of sex-dependent impairments of neuronal function in AD suggests that treatment targeting cell physiology must be adapted differently according to biological sex.

## Supplemental discussion

### Earlier neuronal senescence in NonTg females, compared to NonTg males, is in agreement with an alteration of the axon

The electrophysiological changes seen between old, genetically unmodified males and females were in accordance with axonal dysfunction for many reasons. First, CC is influenced by several factors. An alteration in the myelin of axons is one possibility to explain the increase in CC observed in old females. Myelin sheets are known to decrease the capacitance of covered structures [100]. Consequently, increased CC in female neurons could imply a loss of myelin. Second, an increase in CC without any change in the resistance (for a protocol using hyperpolarizing current) suggests that alterations in cell membranes of old females occur in a structure in which ion channels are inactive for voltages below − 60 mV. Currents involved in AP, the electrical unit transmitted by the axon, include ionic channels activated at voltages above − 60 mV [37], reinforcing the idea that differences between males and females occur at the axon. Third, neurons of old NonTg females demonstrated faster depolarization and repolarization kinetics of APs than males of the same genotype, supporting an axonal difference between the sexes. Changes in myelin sheath organization during aging are known to affect membrane expression of many AP channels [37], suggesting the insertion of additional ones in the axon uncoated region. In sum, our electrophysiological data suggest axonal dysfunction in neurons of NonTg females. Myelin is a major component of white matter and a recent study showed the use of its lipids as a ketogenic fuel supply in aged female mice with a dysfunction in brain energy production [101], suggesting greater susceptibility of females to neuropathologies associated with high energy consumption. Aß peptides are found in a higher level in females and are known to induce brain hyperactivity [34, 52, 102], which could be one pathway explaining their greater susceptibility to AD.

### Transgene expression induces specific alterations in males: a potent factor involved in the differential expression of AD

Release probability of neurotransmitters depends on calcium and on size of the available pool [103]. When two axonal stimulations are evoked in close succession, ratio of the postsynaptic response is a kind of presynaptic plasticity that reflects the probable neurotransmitter release. If presynaptic terminal has higher release probability, the first pulse will deplete available transmitters, and the second one is going to release fewer of them, leading to a low ratio. In return, a low release conducts to a PPR increase since available transmitters remain high and addition of the new calcium entry to the residual calcium from previous AP will induce a higher release of neurotransmitters. Our data demonstrate a lower probability of their release in NonTg male mice, compared to transgenic males. This result is in agreement with studies reporting higher probable neurotransmitter release when an amyloid pathology is present [77]. The higher AP amplitude found in 3xTg-AD males could be a facilitating factor by increasing the depolarization necessary for the activation of calcium channels [37]. This effect of transgenes on PPR has not been seen in females. In a previous study using the same methodology, our laboratory demonstrated that the PPR from NonTg males is similar to that observed in younger mice (≈1.5), while that of old females decreased with aging [50]. Together, these data suggest that aging mechanisms in NonTg female mice decrease PPR by a common mechanism to that of amyloid pathology, thus explaining the lack of transgenic effect in them. In sum, the probability of neurotransmitter release was differently affected in males and females by transgenic expression, and comparison of present results with previous studies performed in younger mice suggests that females exhibit physiological loss of PPR with aging, while men maintain PPR similar to that seen in younger adults when AD is absent.

### Transgene expression induces specific alterations in males: a potent factor involved in the differential expression of AD

The release probability of neurotransmitters depends on calcium and on the size of the available pool [103]. When two axonal stimulations are evoked in close succession, the ratio of the postsynaptic response is a kind of presynaptic plasticity that reflects the probability of neurotransmitters release. If the presynaptic terminal has higher release probability, the first pulse will deplete available transmitters, and the second one will release less transmitters, leading to a low ratio. In contrast, a low release probability conducts to an increase of PPR since the available transmitters remain high and the addition of the new calcium entry to the residual calcium from the previous AP will induce a higher release of neurotransmitters. Our data demonstrate a lower probability of neurotransmitter release in NonTg male mice, compared to transgenic male mice. This result is in agreement with studies reporting a higher probability of neurotransmitter release in the presence of an amyloid pathology [77]. The higher AP amplitude found in 3xTg-AD males could be a facilitating factor by increasing the depolarization necessary for the activation of the calcium channels [37]. This effect of transgenes on PPR has not been observed in females. In a previous study using the same methodology, our laboratory demonstrated that the PPR of NonTg males is similar to that observed in younger mice (≈1.5), while that of old females decreased with aging [50]. Together, these data suggest that the aging mechanisms in NonTg female mice decrease PPR by a common mechanism to that of amyloid pathology, thus explaining the lack of transgenic effect in females. In sum, the probability of neurotransmitter release was differently affected in males and females by transgenic expression and comparison of the present results with previous studies performed in younger mice suggests that females exhibit physiological loss of PPR with aging, while men maintain PPR similar to that seen in younger adults in the absence of AD.

## Supplementary Information

**Additional file 1: Table S1.** Statistical results of the pathological markers. The valuesof each group (t-value / pValue) are separated by a double vertical line (||). *p <0 .05, **p < 0.01 and ***p < 0.001.

**Additional file 2: Table S2.** Statistical results of electrophysiological experiments. The two-way ANOVA shows first effect of the genotype, followed by that of the sex. If variable interaction was detected, statistical comparisons between groups were performed depending on the variance equivalence between groups. An unpaired Student’s t test was performed to compare groups of equal variance whereas groups of data that failed Bartlett's tests of homogeneity of variances were analyzed by Welch’s t test. The values from Student’s / Welch’s t-tests are given accordingly to this order regarding the effect of: (1) transgene expression in females (NonTg females vs. 3xTg-AD females mice); (2) transgene expression in males (NonTg males vs. 3xTg-AD males); (3) sex in NonTg (NonTg males vs. NonTg females); and (4) sex in transgenic animals (3xTg-AD males vs. 3xTg-AD females). The values of each group (t-value / pValue) are separated by a double vertical line (||). Abbreviations: n, number of recorded cells; N, number of mice included in the statistic. *p < 0.05, **p < 0.01 and ***p < 0.001 (effect of transgene expression in animals of the same sex). *p < 0.05, **p < 0.01 and ***p < 0.001 (effect of sex in animals of the same genotype).

**Additional file 3: Table S3.** Statistical results of molecular studies. The two-way ANOVA shows first effect of the genotype, followed by that of the sex. If variable interactions were detected, statistical comparisons between groups were performed depending on the variance equivalence between groups. An unpaired Student’s t test was performed to compare groups of equal variance whereas groups of data that failed tests for equal variance were analyzed by Welch’s t test. The values from Student’s / Welch’s t-tests are given accordingly to this order regarding the effect of: (1) transgene expression in females (NonTg females vs. 3xTg-AD females); (2) transgene expression in males (NonTg male vs. 3xTg-AD males); (3) sex in NonTg (NonTg males vs. NonTg females); and (4) sex in transgenic animals (3xTg-AD males vs. 3xTg-AD females). The values of each group (t-value / pValue) are separated by a double vertical line (||). The two-way ANOVA included three p-values, the effect of genotype (first), sex (second) and variable interaction (third). *p < 0.05 (effect of transgene expression in animals of the same sex). **p < 0.01 (effect of sex in animals of the same genotype).

**Additional file 4: Figure S1.** Original unmodified image of the revelation of GAD65 by western blot in the lysis-buffer soluble fraction.

**Additional file 5: Figure S2.** Original unmodified image of the revelation of actin by western blot in the lysis-buffer soluble fraction. This revelation preceded that of GAD65, which is also present in the image.

**Additional file 6: Figure S3.** Original unmodified image of the revelation of SAP102 by western blot in the TBS soluble fraction.

**Additional file 7: Figure S4.** Original unmodified image of the revelation of actin by western blot in the TBS soluble fraction.

**Additional file 8: Figure S5.** Original unmodified image of the revelation of GAD65 by western blot in the TBS soluble fraction. This revelation preceded that of actin, which is also present in the image.

## Data Availability

All data generated or analyzed during this study are included in this article.

## Abbreviations

3xTg-AD: Triple-transgenic model of AD
AD: Alzheimer’s disease
Aβ: Abeta peptide
AHP: Afterhyperpolarization
AMPA: α-Amino-3-hydroxy-5-methylisoxazole-4-propionic acid
AMPAR: AMPA receptor
AP: Action potential
APP: Amyloid precursor protein
CC: Cell capacitance
EC: Entorhinal cortex
EPSC: Excitatory postsynaptic current
eEPSC: Evoked EPSC
GABAaR: Gamma-aminobutyric acid receptor subunit A
GAD65: Glutamate decarboxylase or glutamic acid decarboxylase
Gc: Cell conductance
Ih: Hyperpolarization-activated cation channel
NMDA: N-methyl-D-aspartate
NonTg: Nontransgenic
PPR: Paired-pulse ratio
PSD-95: Postsynaptic density protein 95 kDa
SAP102: Synapse-associated protein 102
sEPSC: Spontaneous EPSC
VGAT: Vesicular GABA transporter
