# Supplementary figures and images for: Sex-dependent alterations in the physiology of entorhinal cortex neurons in old heterozygous 3xTg-AD mice
Source: Biol Sex Differ. 2020 Nov 16;11:63. doi: 10.1186/s13293-020-00337-0 (PMC7667843; doi:10.1186/s13293-020-00337-0)

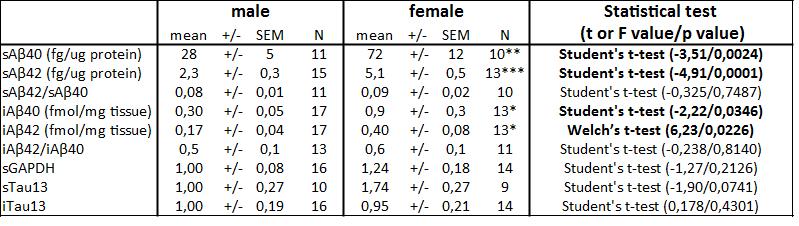

Supplement: Supplementary file 1 — Additional file 1: Table S1. Statistical results of the pathological markers. The valuesof each group (t-value / pValue) are separated by a double vertical line (||). *p <0 .05, **p < 0.01 and ***p < 0.001. [file 13293_2020_337_MOESM1_ESM.jpg]

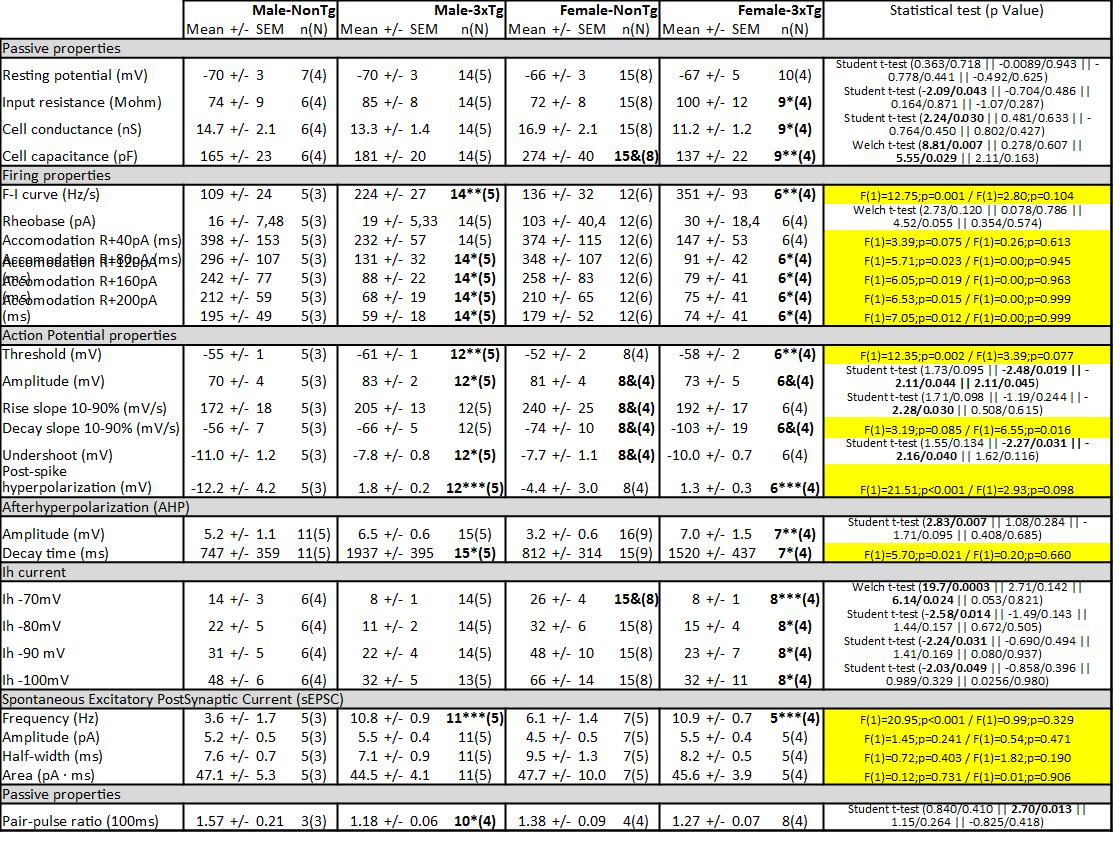

Supplement: Supplementary file 2 — Additional file 2: Table S2. Statistical results of electrophysiological experiments. The two-way ANOVA shows first effect of the genotype, followed by that of the sex. If variable interaction was detected, statistical comparisons between groups were performed depending on the variance equivalence between groups. An unpaired Student’s t test was performed to compare groups of equal variance whereas groups of data that failed Bartlett's tests of homogeneity of variances were analyzed by Welch’s t test. The values from Student’s / Welch’s t-tests are given accordingly to this order regarding the effect of: (1) transgene expression in females (NonTg females vs. 3xTg-AD females mice); (2) transgene expression in males (NonTg males vs. 3xTg-AD males); (3) sex in NonTg (NonTg males vs. NonTg females); and (4) sex in transgenic animals (3xTg-AD males vs. 3xTg-AD females). The values of each group (t-value / pValue) are separated by a double vertical line (||). Abbreviations: n, number of recorded cells; N, number of mice included in the statistic. *p < 0.05, **p < 0.01 and ***p < 0.001 (effect of transgene expression in animals of the same sex). *p < 0.05, **p < 0.01 and ***p < 0.001 (effect of sex in animals of the same genotype). [file 13293_2020_337_MOESM2_ESM.jpg]

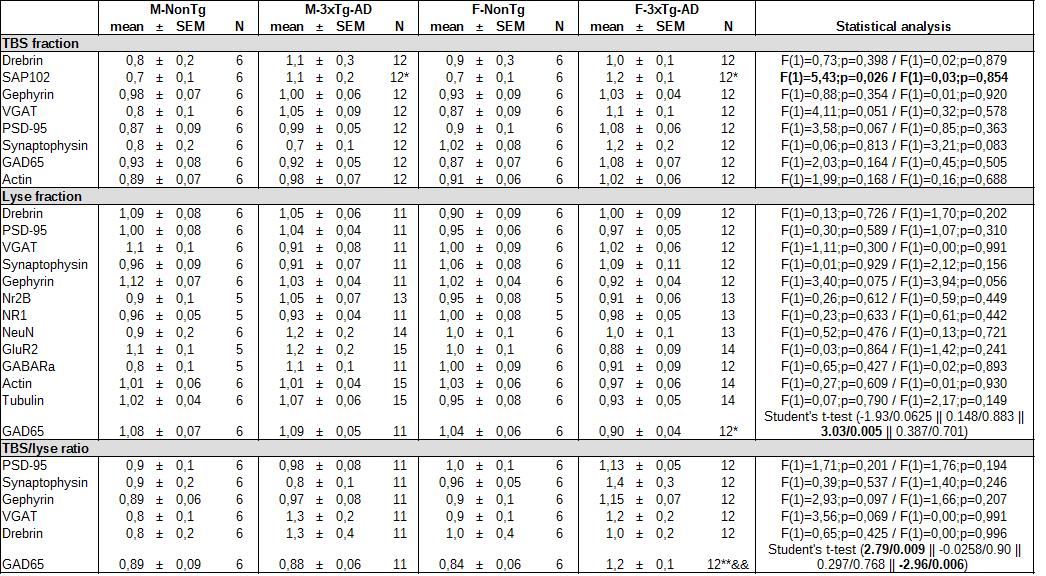

Supplement: Supplementary file 3 — Additional file 3: Table S3. Statistical results of molecular studies. The two-way ANOVA shows first effect of the genotype, followed by that of the sex. If variable interactions were detected, statistical comparisons between groups were performed depending on the variance equivalence between groups. An unpaired Student’s t test was performed to compare groups of equal variance whereas groups of data that failed tests for equal variance were analyzed by Welch’s t test. The values from Student’s / Welch’s t-tests are given accordingly to this order regarding the effect of: (1) transgene expression in females (NonTg females vs. 3xTg-AD females); (2) transgene expression in males (NonTg male vs. 3xTg-AD males); (3) sex in NonTg (NonTg males vs. NonTg females); and (4) sex in transgenic animals (3xTg-AD males vs. 3xTg-AD females). The values of each group (t-value / pValue) are separated by a double vertical line (||). The two-way ANOVA included three p-values, the effect of genotype (first), sex (second) and variable interaction (third). *p < 0.05 (effect of transgene expression in animals of the same sex). **p < 0.01 (effect of sex in animals of the same genotype). [file 13293_2020_337_MOESM3_ESM.jpg]

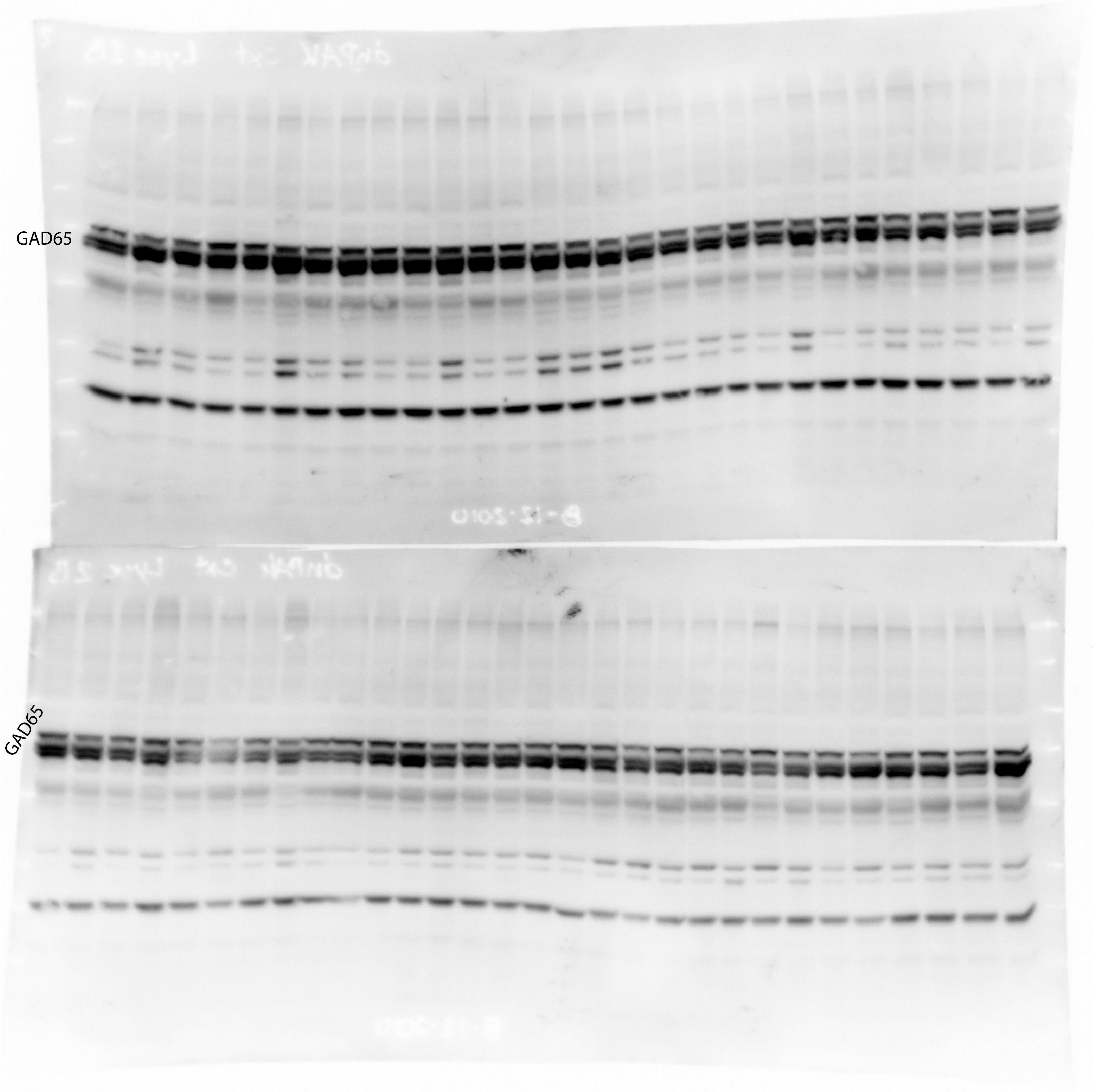

Supplement: Supplementary file 4 — Additional file 4: Figure S1. Original unmodified image of the revelation of GAD65 by western blot in the lysis-buffer soluble fraction. [file 13293_2020_337_MOESM4_ESM.jpg]

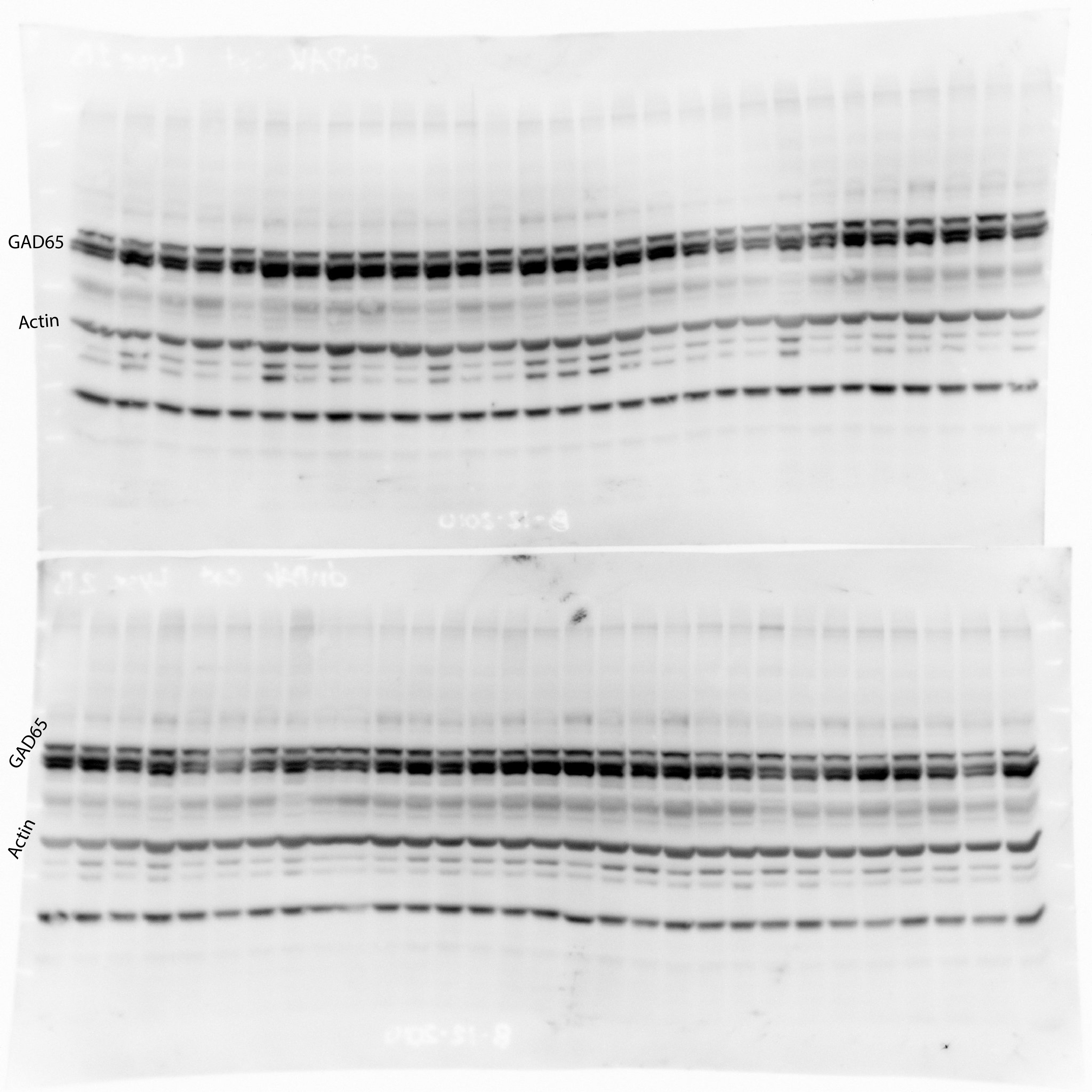

Supplement: Supplementary file 5 — Additional file 5: Figure S2. Original unmodified image of the revelation of actin by western blot in the lysis-buffer soluble fraction. This revelation preceded that of GAD65, which is also present in the image. [file 13293_2020_337_MOESM5_ESM.jpg]

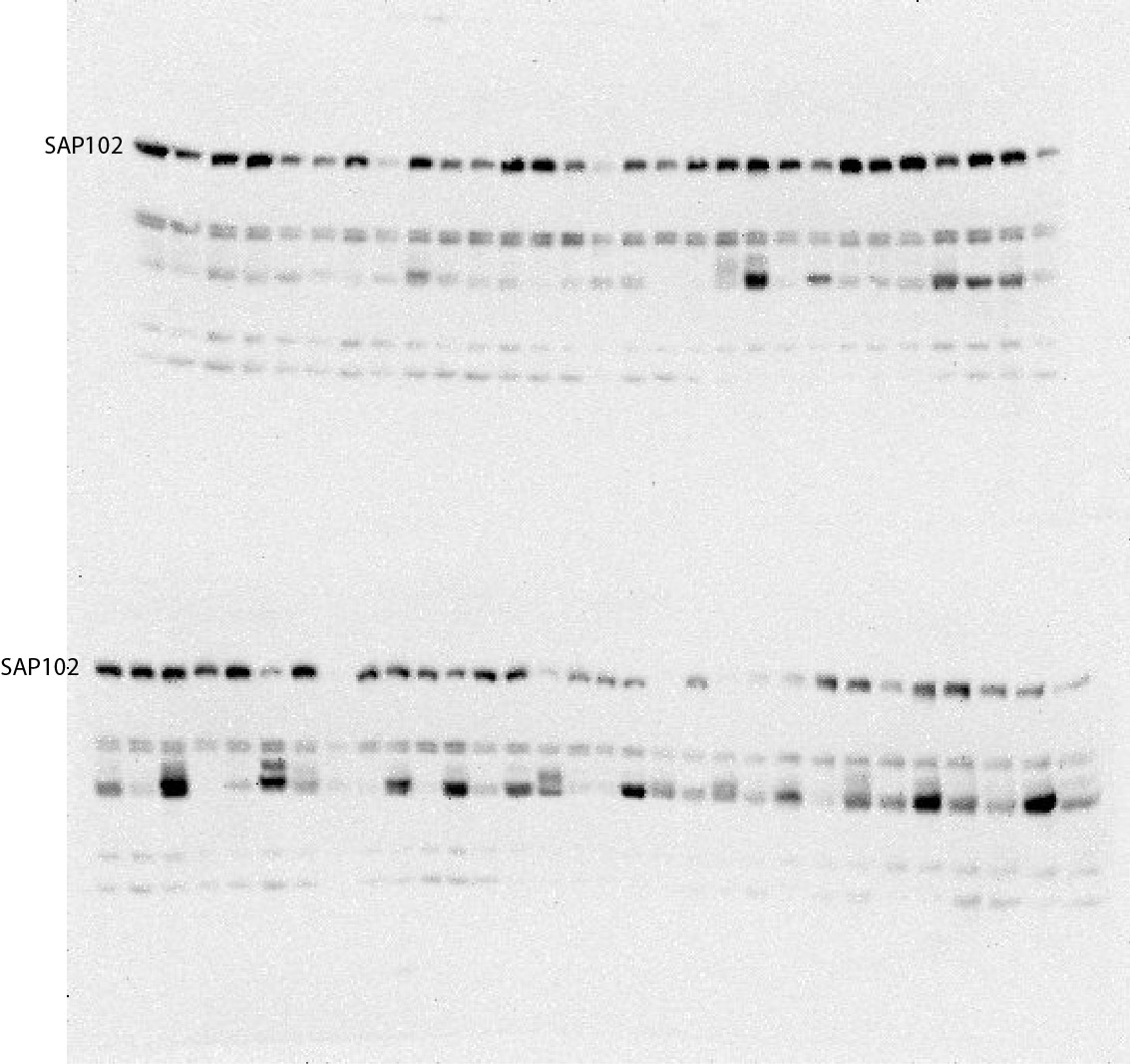

Supplement: Supplementary file 6 — Additional file 6: Figure S3. Original unmodified image of the revelation of SAP102 by western blot in the TBS soluble fraction. [file 13293_2020_337_MOESM6_ESM.jpg]

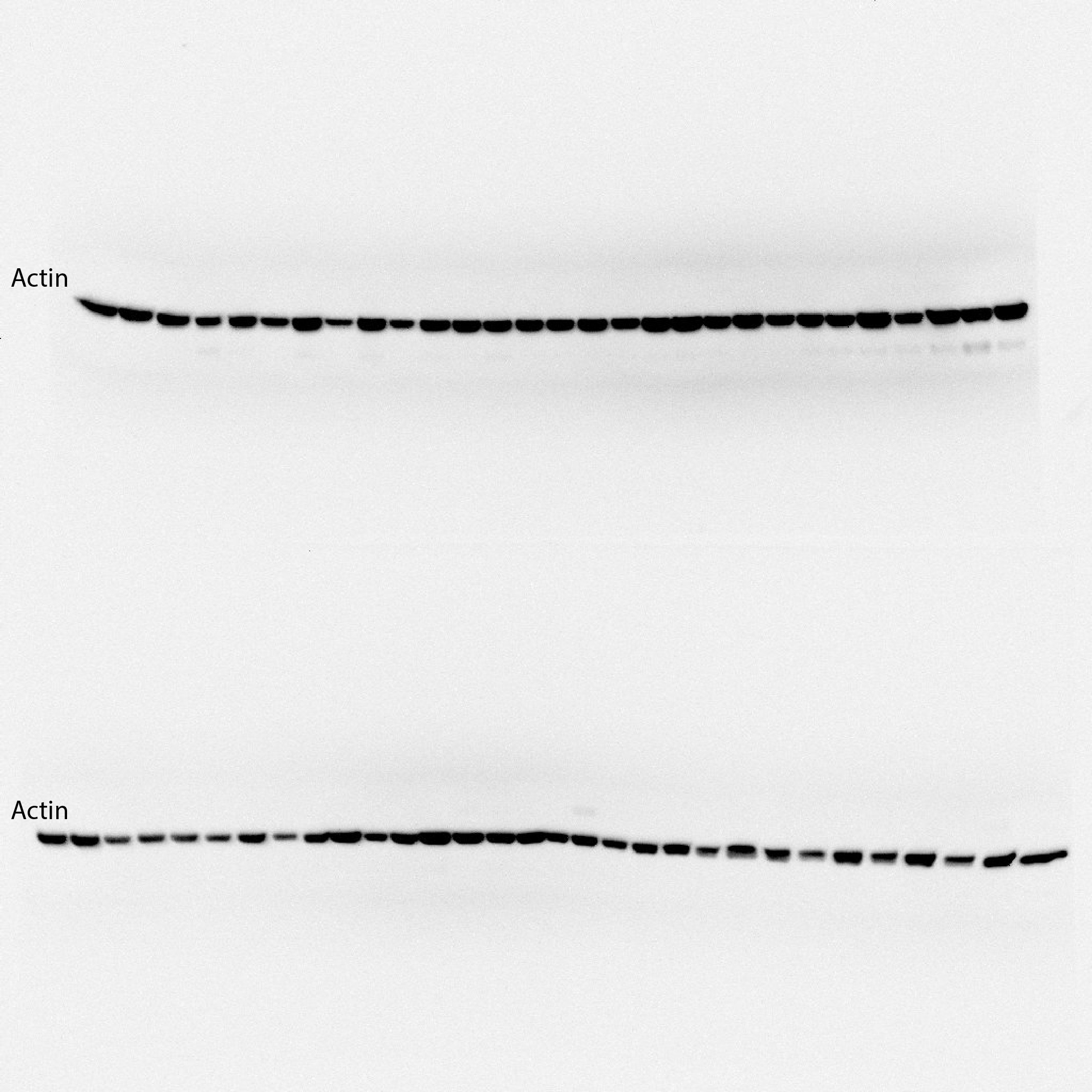

Supplement: Supplementary file 7 — Additional file 7: Figure S4. Original unmodified image of the revelation of actin by western blot in the TBS soluble fraction. [file 13293_2020_337_MOESM7_ESM.jpg]

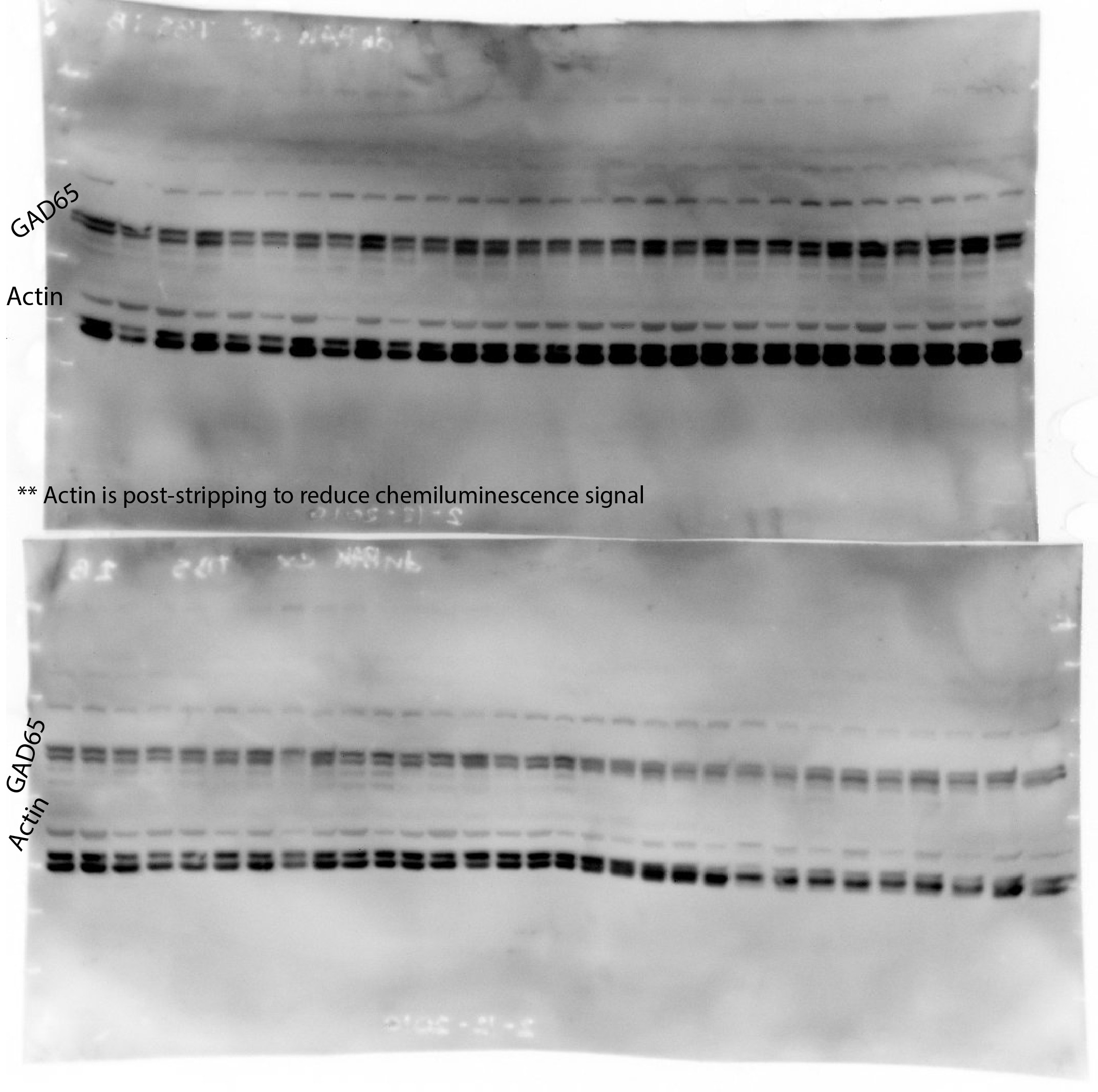

Supplement: Supplementary file 8 — Additional file 8: Figure S5. Original unmodified image of the revelation of GAD65 by western blot in the TBS soluble fraction. This revelation preceded that of actin, which is also present in the image. [file 13293_2020_337_MOESM8_ESM.jpg]
